# An algebra of reversible computation

**DOI:** 10.1186/s40064-016-3229-7

**Published:** 2016-09-26

**Authors:** Yong Wang

**Affiliations:** College of Computer Science, Beijing University of Technology, Beijing, China

**Keywords:** Reversible computation, Process algebra, Algebra of communicating processes, Axiomatization

## Abstract

We design an axiomatization for reversible computation called reversible ACP (RACP). It has four extendible modules: basic reversible processes algebra, algebra of reversible communicating processes, recursion and abstraction. Just like process algebra ACP in classical computing, RACP can be treated as an axiomatization foundation for reversible computation.

## Background

Reversible computation (Perumalla 2013) has gained more and more attention in many application areas, such as the modeling of biochemical systems, program debugging and testing, and also quantum computing. For the excellent properties reversible computing has, it will be exploited in many computing devices in the future.

There are several research works on reversible computation. Abramsky maps functional programs into reversible automata (Abramsky 2005). Danos and Krivine’s reversible RCCS (Danos and Krivine 2005) uses the concept of thread to reverse a CCS (Milner 1989; Milner et al. 1992) process. Reversible CCS (RCCS) has been proposed as a first causal-consistent reversible calculus. It introduces the idea of attaching memories to threads in order to keep the history of the computation. Boudol and Castellani (1988, 1994) compare three different non-interleaving models for CCS: proved transition systems, event structures and Petri nets. Phillips and Ulidowski’s CCSK (Phillips 2007; Ulidowski et al. 2014; Phillips and Ulidowski 2012) formulates a procedure for converting operators of standard algebraic process calculi such as CCS into reversible operators, while preserving their operational semantics. CCSK defines the so-called forward–reverse bisimulation and show that it is preserved by all reversible operators. CCSK is the extension of CCS for a general reversible process calculus. The main novelty of CCSK is that the structure of processes is not consumed, but simply annotated when they are executed. This is obtained by making all the rules defining the semantics static. Thus, no memories are needed. And other efforts on reversible computations, such as reversibility on pi (Lanese et al. 2010, 2011, 2013), reversibility and compensation (Lanese et al. 2012), reversibility and fault-tolerances (Perumalla and Park 2013), and reversibility in massive concurrent systems (Cardelli and Laneve 2011). And the recently quantitative analysis of concurrent reversible computations (Marin and Rossi 2015).

In process algebra (Baeten 2005), ACP (Fokkink 2007) can be treated as a refinement of CCS (Milner 1989; Milner et al. 1992). CCSK uses the so-called communication key to mark the histories of an atomic action (called past action) and remains the structural operational semantics. We are inspired by the way of CCSK: is there an axiomatic algebra to refine CCSK, just like the relation to ACP and CCS? We do it along the way paved by CCSK and ACP, and lead to a new reversible axiomatic algebra, we called it as reversible ACP (RACP).

RACP is an axiomatic refinement to CCSK:

1. It has more concise structural operation semantics for forward transitions and reverse transitions, without more predicates, such as standard process predicate and freshness predicate.
2. It has four extendible modules, basic reversible processes algebra (BRPA), algebra of reversible communicating processes (ARCP), recursion and abstraction. While in CCSK, recursion and abstraction are not concerned.
3. In comparison to ACP, it is almost a brand new algebra for reversible computation which has the same advantages of ACP, such as modularity, axiomatization, etc. Firstly, in RACP, the alternative composition is replaced by choice composition, since in reversible computing, all choice branches should be retained. Secondly, the parallel operator cannot be captured by an interleaving semantics. Thirdly, more importantly to establish a full axiomatization, all the atomic actions are distinct, the same atomic action in different branches (including choice branches and parallel branches) will be deemed as the same **one** atomic action. Also auto-concurrency is out of scope for our work here.

The paper is organized as follows. In section “Preliminaries”, some basic concepts related to equational logic, structural operational semantics and process algebra ACP are introduced. The BRPA is introduced in section “BRPA: basic reversible process algebra”, ARCP is introduced in section “ARCP: algebra of reversible communicating processes”, recursion is introduced in section “Recursion”, and abstraction is introduced in section “Abstraction”. An application of RACP is introduced in section “Verification for business protocols with compensation support”. We discuss the extensions of RACP in section “Extensions”. Finally, we conclude this paper in section “Conclusions”.

## Preliminaries

For convenience of the reader, we introduce some basic concepts about equational logic, structural operational semantics and process algebra ACP (please refer to Plotkin 1981, Fokkink 2007 for more details).

### Equational logic

We introduce some basic concepts related to equational logic briefly, including signature, term, substitution, axiomatization, equality relation, model, term rewriting system, rewrite relation, normal form, termination, weak confluence and several conclusions. These concepts originate from Fokkink (2007), and are introduced briefly as follows. About the details, please see Fokkink (2007).

#### **Definition 1**

(*Signature*) A signature $\varSigma$ consists of a finite set of function symbols (or operators) $f,g,\ldots$, where each function symbol *f* has an arity *ar*(*f*), being its number of arguments. A function symbol *a*, *b*, *c*, …of arity *zero* is called a constant, a function symbol of arity one is called unary, and a function symbol of arity two is called binary.

#### **Definition 2**

(*Term*) Let $\varSigma$ be a signature. The set ${\mathbb {T}}(\varSigma )$ of (open) terms *s*, *t*, *u*, …over $\varSigma$ is defined as the least set satisfying: (1) each variable is in ${\mathbb {T}}(\varSigma )$; (2) if $f\in \varSigma$ and $t_1,\ldots ,t_{ar(f)}\in {\mathbb {T}}(\varSigma )$, then $f(t_1,\ldots ,t_{ar(f)}\in {\mathbb {T}}(\varSigma ))$. A term is closed if it does not contain variables. The set of closed terms is denoted by ${\mathcal {T}}(\varSigma )$.

#### **Definition 3**

(*Substitution*) Let $\varSigma$ be a signature. A substitution is a mapping $\sigma$ from variables to the set ${\mathbb {T}}(\varSigma )$ of open terms. A substitution extends to a mapping from open terms to open terms: the term $\sigma (t)$ is obtained by replacing occurrences of variables *x* in t by $\sigma (x)$. A substitution $\sigma$ is closed if $\sigma (x)\in {\mathcal {T}}(\varSigma )$ for all variables *x*.

#### **Definition 4**

(*Axiomatization*) An axiomatization over a signature $\varSigma$ is a finite set of equations, called axioms, of the form $s=t$ with $s,t\in {\mathbb {T}}(\varSigma )$.

#### **Definition 5**

(*Equality relation*) An axiomatization over a signature $\varSigma$ induces a binary equality relation $=$ on ${\mathbb {T}}(\varSigma )$ as follows. (1) (Substitution) If $s=t$ is an axiom and $\sigma$ a substitution, then $\sigma (s)=\sigma (t)$. (2) (Equivalence) The relation = is closed under reflexivity, symmetry, and transitivity. (3) (Context) The relation = is closed under contexts: if $t=u$ and *f* is a function symbol with $ar(f)>0$, then $f(s_1,\ldots ,s_{i-1},t,s_{i+1}, \ldots ,s_{ar(f)})=f(s_1,\ldots ,s_{i-1},u,s_{i+1},\ldots ,s_{ar(f)})$.

#### **Definition 6**

(*Model*) Assume an axiomatization ${\mathcal {E}}$ over a signature $\varSigma$, which induces an equality relation =. A model for ${\mathcal {E}}$ consists of a set ${\mathcal {M}}$ together with a mapping $\phi : {\mathcal {T}}(\varSigma )\rightarrow {\mathcal {M}}$. (1) $({\mathcal {M}},\phi )$ is sound for ${\mathcal {E}}$ if $s=t$ implies $\phi (s)\equiv \phi (t)$ for $s,t\in {\mathcal {T}}(\varSigma )$; (2) $({\mathcal {M}},\phi )$ is complete for ${\mathcal {E}}$ if $\phi (s)\equiv \phi (t)$ implies $s=t$ for $s,t\in {\mathcal {T}}(\varSigma )$.

#### **Definition 7**

(*Term rewriting system*) Assume a signature $\varSigma$. A rewrite rule is an expression $s\rightarrow t$ with $s,t\in {\mathbb {T}}(\varSigma )$, where: (1) the left-hand side *s* is not a single variable; (2) all variables that occur at the right-hand side *t* also occur in the left-hand side *s*. A term rewriting system (TRS) is a finite set of rewrite rules.

#### **Definition 8**

(*Rewrite relation*) A TRS over a signature $\varSigma$ induces a one-step rewrite relation $\rightarrow$ on ${\mathbb {T}}(\varSigma )$ as follows. (1) (Substitution) If $s\rightarrow t$ is a rewrite rule and $\sigma$ a substitution, then $\sigma (s)\rightarrow \sigma (t)$. (2) (Context) The relation $\rightarrow$ is closed under contexts: if $t\rightarrow u$ and *f* is a function symbol with $ar(f)>0$, then $f(s_1,\ldots ,s_{i-1},t,s_{i+1},\ldots ,s_{ar(f)})\rightarrow f(s_1,\ldots ,s_{i-1},u,s_{i+1},\ldots ,s_{ar(f)})$. The rewrite relation $\rightarrow ^*$ is the reflexive transitive closure of the one-step rewrite relation $\rightarrow$: (1) if $s\rightarrow t$, then $s\rightarrow ^* t$; (2) $t\rightarrow ^* t$; (3) if $s\rightarrow ^* t$ and $t\rightarrow ^* u$, then $s\rightarrow ^* u$.

#### **Definition 9**

(*Normal form*) A term is called a normal form for a TRS if it cannot be reduced by any of the rewrite rules.

#### **Definition 10**

(*Termination*) A TRS is terminating if it does not induce infinite reductions $t_0\rightarrow t_1\rightarrow t_2\rightarrow \cdots$.

#### **Definition 11**

(*Weak confluence*) A TRS is weakly confluent if for each pair of one-step reductions $s\rightarrow t_1$ and $s\rightarrow t_2$, there is a term *u* such that $t_1\rightarrow ^* u$ and $t_2\rightarrow ^* u$.

#### **Theorem 1**

(Newman’s lemma)* If a TRS is terminating and weakly confluent, then it reduces each term to a unique normal form.*

#### **Definition 12**

(*Commutativity and associativity*) Assume an axiomatization ${\mathcal {E}}$. A binary function symbol *f* is commutative if ${\mathcal {E}}$ contains an axiom $f(x,y)=f(y,x)$ and associative if ${\mathcal {E}}$ contains an axiom $f(f(x,y),z)=f(x,f(y,z))$.

#### **Definition 13**

(*Convergence*) A pair of terms *s* and *t* is said to be convergent if there exists a term *u* such that $s\rightarrow ^* u$ and $t\rightarrow ^* u$.

Axiomatizations can give rise to TRSs that are not weakly confluent, which can be remedied by Knuth–Bendix completion (Knuth and Bendix 1970). It determines overlaps in left hand sides of rewrite rules, and introduces extra rewrite rules to join the resulting right hand sides, which are called critical pairs.

#### **Theorem 2**

*A TRS is weakly confluent if and only if all its critical pairs are convergent.*

### Structural operational semantics

The concepts about structural operational semantics include labelled transition system (LTS), transition system specification (TSS), transition rule and its source, source-dependent, conservative extension, fresh operator, panth format, congruence, bisimulation, etc. These concepts are coming from Fokkink (2007), and are introduced briefly as follows. About the details, please see Plotkin (1981). Also, to support reversible computation, we introduce a new kind of bisimulation called forward–reverse bisimulation (FR bisimulation) which occurred in De Nicola et al. (1990) and Phillips (2007).

We assume a non-empty set *S* of states, a finite, non-empty set of transition labels *A* and a finite set of predicate symbols.

#### **Definition 14**

(*Labeled transition system*) A transition is a triple $(s,a,s')$ with $a\in A$, or a pair (*s*, *P*) with *P* a predicate, where $s,s'\in S$. A labeled transition system (LTS) is possibly infinite set of transitions. An LTS is finitely branching if each of its states has only finitely many outgoing transitions.

#### **Definition 15**

(*Transition system specification*) A transition rule $\rho$ is an expression of the form $\frac{H}{\pi }$, with *H* a set of expressions $t\xrightarrow {a}t'$ and *tP* with $t,t'\in {\mathbb {T}}(\varSigma )$, called the (positive) premises of $\rho$, and $\pi$ an expression $t\xrightarrow {a}t'$ or *tP* with $t,t'\in {\mathbb {T}}(\varSigma )$, called the conclusion of $\rho$. The left-hand side of $\pi$ is called the source of $\rho$. A transition rule is closed if it does not contain any variables. A transition system specification (TSS) is a (possible infinite) set of transition rules.

#### **Definition 16**

(*Proof*) A proof from a TSS *T* of a closed transition rule $\frac{H}{\pi }$ consists of an upwardly branching tree in which all upward paths are finite, where the nodes of the tree are labelled by transitions such that: (1) the root has label $\pi$; (2) if some node has label *l*, and *K* is the set of labels of nodes directly above this node, then (a) either *K* is the empty set and $l\in H$, (b) or $\frac{K}{l}$ is a closed substitution instance of a transition rule in *T*.

#### **Definition 17**

(*Generated LTS*) We define that the LTS generated by a TSS *T* consists of the transitions $\pi$ such that $\frac{\emptyset }{\pi }$ can be proved from *T*.

#### **Definition 18**

A set *N* of expressions $t\nrightarrow ^{a}$ and $t\lnot P$ (where *t* ranges over closed terms, *a* over *A* and *P* over predicates) hold for a set ${\mathcal {S}}$ of transitions, denoted by ${\mathcal {S}}\vDash N$, if: (1) for each $t\nrightarrow ^{a} \in N$ we have that $t\xrightarrow {a}t' \notin {\mathcal {S}}$ for all $t'\in {\mathcal {T}}(\varSigma )$; (2) for each $t\lnot P\in N$ we have that $tP \notin {\mathcal {S}}$.

#### **Definition 19**

(*Three-valued stable model*) A pair $\langle {\mathcal {C}},\mathcal {U}\rangle$ of disjoint sets of transitions is a three-valued stable model for a TSS *T* if it satisfies the following two requirements: (1) a transition $\pi$ is in ${\mathcal {C}}$ if and only if *T* proves a closed transition rule $\frac{N}{\pi }$ where *N* contains only negative premises and ${\mathcal {C}}\cup \mathcal {U}\vDash N$; (2) a transition $\pi$ is in ${\mathcal {C}}\cup \mathcal {U}$ if and only if *T* proves a closed transition rule $\frac{N}{\pi }$ where *N* contains only negative premises and ${\mathcal {C}}\vDash N$.

#### **Definition 20**

(*Ordinal number*) The ordinal numbers are defined inductively by: (1) 0 is the smallest ordinal number; (2) each ordinal number $\alpha$ has a successor $\alpha + 1$; (3) each sequence of ordinal number $\alpha< \alpha + 1< \alpha + 2 < \cdots$ is capped by a limit ordinal $\lambda$.

#### **Definition 21**

(*Positive after reduction*) A TSS is positive after reduction if its least three-valued stable model does not contain unknown transitions.

#### **Definition 22**

(*Stratification*) A stratification for a TSS is a weight function $\phi$ which maps transitions to ordinal numbers, such that for each transition rule $\rho$ with conclusion $\pi$ and for each closed substitution $\sigma$: (1) for positive premises $t\xrightarrow {a}t'$ and *tP* of $\rho , \phi (\sigma (t)\xrightarrow {a}\sigma (t'))\le \phi (\sigma (\pi ))$ and $\phi (\sigma (t)P\le \phi (\sigma (\pi )))$, respectively; (2) for negative premise $t\nrightarrow ^{a}$ and $t\lnot P$ of $\rho , \phi (\sigma (t)\xrightarrow {a}t')< \phi (\sigma (\pi ))$ for all closed terms $t'$ and $\phi (\sigma (t)P < \phi (\sigma (\pi )))$, respectively.

#### **Theorem 3**

*If a TSS allows a stratification, then it is positive after reduction.*

#### **Definition 23**

(*Process graph*) A process (graph) *p* is an LTS in which one state *s* is elected to be the root. If the LTS contains a transition $s\xrightarrow {a} s'$, then $p\xrightarrow {a} p'$ where $p'$ has root state $s'$. Moreover, if the LTS contains a transition *sP*, then *pP*. (1) A process $p_0$ is finite if there are only finitely many sequences $p_0\xrightarrow {a_1}p_1\xrightarrow {a_2}\cdots \xrightarrow {a_k} P_k$. (2) A process $p_0$ is regular if there are only finitely many processes $p_k$ such that $p_0\xrightarrow {a_1}p_1\xrightarrow {a_2}\cdots \xrightarrow {a_k} P_k$.

#### **Definition 24**

(*Reverse transition*) There are two processes *p* and $p'$, two transitions $p \xrightarrow {a} p'$ and $p' \mathop {\twoheadrightarrow}{a[m]} p$, the transition $p' \mathop {\twoheadrightarrow}{a[m]} p$ is called reverse transition of $p \xrightarrow {a} p'$, and the transition $p \xrightarrow {a} p'$ is called forward transition. If $p \xrightarrow {a} p'$ then $p' \mathop {\twoheadrightarrow}{a[m]} p$, the forward transition $p \xrightarrow {a} p'$ is reversible. Where *a*[*m*] is a kind of special action constant $a[m]\in A \times {\mathcal {K}}, {\mathcal {K}}\subseteq {\mathbb {N}}$, called the histories of an action *a*, and $m\in {\mathcal {K}}$.

#### **Definition 25**

(*Bisimulation*) A bisimulation relation ${\mathcal {B}}$ is a binary relation on processes such that: (1) if $p{\mathcal {B}}q$ and $p\xrightarrow {a}p'$ then $q\xrightarrow {a}q'$ with $p'{\mathcal {B}}q'$; (2) if $p{\mathcal {B}}q$ and $q\xrightarrow {a}q'$ then $p\xrightarrow {a}p'$ with $p'{\mathcal {B}}q'$; (3) if $p{\mathcal {B}}q$ and *pP*, then *qP*; (4) if $p{\mathcal {B}}q$ and *qP*, then *pP*. Two processes *p* and *q* are bisimilar, denoted by $p\underline{\leftrightarrow } q$, if there is a bisimulation relation ${\mathcal {B}}$ such that $p{\mathcal {B}}q$.

#### **Definition 26**

(*Forward–reverse bisimulation*) A forward–reverse (FR) bisimulation relation ${\mathcal {B}}$ is a binary relation on processes such that: (1) if $p{\mathcal {B}}q$ and $p\xrightarrow {a}p'$ then $q\xrightarrow {a}q'$ with $p'{\mathcal {B}}q'$; (2) if $p{\mathcal {B}}q$ and $q\xrightarrow {a}q'$ then $p\xrightarrow {a}p'$ with $p'{\mathcal {B}}q'$; (3)if $p{\mathcal {B}}q$ and $p\mathop {\twoheadrightarrow}{a[m]}p'$ then $q\mathop {\twoheadrightarrow}{a[m]}q'$ with $p'{\mathcal {B}}q'$; (4) if $p{\mathcal {B}}q$ and $q\mathop {\twoheadrightarrow}{a[m]}q'$ then $p\mathop {\twoheadrightarrow}{a[m]}p'$ with $p'{\mathcal {B}}q'$; (5) if $p{\mathcal {B}}q$ and *pP*, then *qP*; (6) if $p{\mathcal {B}}q$ and *qP*, then *pP*. Two processes *p* and *q* are FR bisimilar, denoted by $p\underline{\leftrightarrow }^{fr} q$, if there is a FR bisimulation relation ${\mathcal {B}}$ such that $p{\mathcal {B}}q$.

#### **Definition 27**

(*Congruence*) Let $\varSigma$ be a signature. An equivalence relation ${\mathcal {B}}$ on ${\mathcal {T}}(\varSigma )$ is a congruence if for each $f\in \varSigma$, if $s_i{\mathcal {B}}t_i$ for $i\in \{1,\ldots ,ar(f)\}$, then $f(s_1,\ldots ,s_{ar(f)}){\mathcal {B}}f(t_1,\ldots ,t_{ar(f)})$.

#### **Definition 28**

(*Panth format*) A transition rule $\rho$ is in panth format if it satisfies the following three restrictions: (1) for each positive premise $t\xrightarrow {a} t'$ of $\rho$, the right-hand side $t'$ is single variable; (2) the source of $\rho$ contains no more than one function symbol; (3) there are no multiple occurrences of the same variable at the right-hand sides of positive premises and in the source of $\rho$. A TSS is said to be in panth format if it consists of panth rules only.

#### **Theorem 4**

*If a TSS is positive after reduction and in panth format, then the bisimulation equivalence that it induces is a congruence.*

#### **Definition 29**

(*Branching bisimulation*) A branching bisimulation relation ${\mathcal {B}}$ is a binary relation on the collection of processes such that: (1) if $p{\mathcal {B}}q$ and $p\xrightarrow {a}p'$ then either $a\equiv \tau$ and $p'{\mathcal {B}}q$ or there is a sequence of (zero or more) $\tau$-transitions $q\xrightarrow {\tau }\cdots \xrightarrow {\tau }q_0$ such that $p{\mathcal {B}}q_0$ and $q_0\xrightarrow {a}q'$ with $p'{\mathcal {B}}q'$; (2) if $p{\mathcal {B}}q$ and $q\xrightarrow {a}q'$ then either $a\equiv \tau$ and $p{\mathcal {B}}q'$ or there is a sequence of (zero or more) $\tau$-transitions $p\xrightarrow {\tau }\cdots \xrightarrow {\tau }p_0$ such that $p_0{\mathcal {B}}q$ and $p_0\xrightarrow {a}p'$ with $p'{\mathcal {B}}q'$; (3) if $p{\mathcal {B}}q$ and *pP*, then there is a sequence of (zero or more) $\tau$-transitions $q\xrightarrow {\tau }\cdots \xrightarrow {\tau }q_0$ such that $p{\mathcal {B}}q_0$ and $q_0P$; (4) if $p{\mathcal {B}}q$ and *qP*, then there is a sequence of (zero or more) $\tau$-transitions $p\xrightarrow {\tau }\cdots \xrightarrow {\tau }p_0$ such that $p_0{\mathcal {B}}q$ and $p_0P$. Two processes *p* and *q* are branching bisimilar, denoted by $p\underline{\leftrightarrow }_b q$, if there is a branching bisimulation relation ${\mathcal {B}}$ such that $p{\mathcal {B}}q$.

#### **Definition 30**

(*Branching forward–reverse bisimulation*) A branching forward–reverse (FR) bisimulation relation ${\mathcal {B}}$ is a binary relation on the collection of processes such that: (1) if $p{\mathcal {B}}q$ and $p\xrightarrow {a}p'$ then either $a\equiv \tau$ and $p'{\mathcal {B}}q$ or there is a sequence of (zero or more) $\tau$-transitions $q\xrightarrow {\tau }\cdots \xrightarrow {\tau }q_0$ such that $p{\mathcal {B}}q_0$ and $q_0\xrightarrow {a}q'$ with $p'{\mathcal {B}}q'$; (2) if $p{\mathcal {B}}q$ and $q\xrightarrow {a}q'$ then either $a\equiv \tau$ and $p{\mathcal {B}}q'$ or there is a sequence of (zero or more) $\tau$-transitions $p\xrightarrow {\tau }\cdots \xrightarrow {\tau }p_0$ such that $p_0{\mathcal {B}}q$ and $p_0\xrightarrow {a}p'$ with $p'{\mathcal {B}}q'$; (3) if $p{\mathcal {B}}q$ and *pP*, then there is a sequence of (zero or more) $\tau$-transitions $q\xrightarrow {\tau }\cdots \xrightarrow {\tau }q_0$ such that $p{\mathcal {B}}q_0$ and $q_0P$; (4) if $p{\mathcal {B}}q$ and *qP*, then there is a sequence of (zero or more) $\tau$-transitions $p\xrightarrow {\tau }\cdots \xrightarrow {\tau }p_0$ such that $p_0{\mathcal {B}}q$ and $p_0P$; (5) if $p{\mathcal {B}}q$ and $p\mathop {\twoheadrightarrow}{a[m]}p'$ then either $a\equiv \tau$ and $p'{\mathcal {B}}q$ or there is a sequence of (zero or more) $\tau$-transitions $q\mathop {\twoheadrightarrow }\limits ^{\tau }\ldots \mathop {\twoheadrightarrow }\limits ^{\tau }q_0$ such that $p{\mathcal {B}}q_0$ and $q_0\mathop {\twoheadrightarrow}{a[m]}q'$ with $p'{\mathcal {B}}q'$; (6) if $p{\mathcal {B}}q$ and $q\mathop {\twoheadrightarrow}{a[m]}q'$ then either $a\equiv \tau$ and $p{\mathcal {B}}q'$ or there is a sequence of (zero or more) $\tau$-transitions $p\mathop {\twoheadrightarrow }\limits ^{\tau }\ldots \mathop {\twoheadrightarrow }\limits ^{\tau }p_0$ such that $p_0{\mathcal {B}}q$ and $p_0\mathop {\twoheadrightarrow}{a[m]}p'$ with $p'{\mathcal {B}}q'$; (7) if $p{\mathcal {B}}q$ and *pP*, then there is a sequence of (zero or more) $\tau$-transitions $q\mathop {\twoheadrightarrow }\limits ^{\tau }\ldots \mathop {\twoheadrightarrow }\limits ^{\tau }q_0$ such that $p{\mathcal {B}}q_0$ and $q_0P$; (8) if $p{\mathcal {B}}q$ and *qP*, then there is a sequence of (zero or more) $\tau$-transitions $p\mathop {\twoheadrightarrow }\limits ^{\tau }\ldots \mathop {\twoheadrightarrow }\limits ^{\tau }p_0$ such that $p_0{\mathcal {B}}q$ and $p_0P$. Two processes *p* and *q* are branching FR bisimilar, denoted by $p{\underline{\leftrightarrow }}^{fr}_b q$, if there is a branching FR bisimulation relation ${\mathcal {B}}$ such that $p{\mathcal {B}}q$.

#### **Definition 31**

(*Rooted branching bisimulation*) A rooted branching bisimulation relation ${\mathcal {B}}$ is a binary relation on processes such that: (1) if $p{\mathcal {B}}q$ and $p\xrightarrow {a}p'$ then $q\xrightarrow {a}q'$ with $p'\underline{\leftrightarrow }_b q'$; (2) if $p{\mathcal {B}}q$ and $q\xrightarrow {a}q'$ then $p\xrightarrow {a}p'$ with $p'\underline{\leftrightarrow }_b q'$; (3) if $p{\mathcal {B}}q$ and *pP*, then *qP*; (4) if $p{\mathcal {B}}q$ and *qP*, then *pP*. Two processes *p* and *q* are rooted branching bisimilar, denoted by $p\underline{\leftrightarrow }_{rb} q$, if there is a rooted branching bisimulation relation ${\mathcal {B}}$ such that $p{\mathcal {B}}q$.

#### **Definition 32**

(*Rooted branching forward–reverse bisimulation*) A rooted branching forward–reverse (FR) bisimulation relation ${\mathcal {B}}$ is a binary relation on processes such that: (1) if $p{\mathcal {B}}q$ and $p\xrightarrow {a}p'$ then $q\xrightarrow {a}q'$ with $p'{\underline{\leftrightarrow }}^{fr}_b q'$; (2) if $p{\mathcal {B}}q$ and $q\xrightarrow {a}q'$ then $p\xrightarrow {a}p'$ with $p'{\underline{\leftrightarrow }}^{fr}_b q'$; (3) if $p{\mathcal {B}}q$ and $p\mathop {\twoheadrightarrow}{a[m]}p'$ then $q\mathop {\twoheadrightarrow}{a[m]}q'$ with $p'{\underline{\leftrightarrow }}^{fr}_b q'$; (4) if $p{\mathcal {B}}q$ and $q\mathop {\twoheadrightarrow}{a[m]}q'$ then $p\mathop {\twoheadrightarrow}{a[m]}p'$ with $p'{\underline{\leftrightarrow }}^{fr}_b q'$; (5) if $p{\mathcal {B}}q$ and *pP*, then *qP*; (6) if $p{\mathcal {B}}q$ and *qP*, then *pP*. Two processes *p* and *q* are rooted branching FR bisimilar, denoted by $p{\underline{\leftrightarrow }}^{fr}_{rb} q$, if there is a rooted branching FR bisimulation relation ${\mathcal {B}}$ such that $p{\mathcal {B}}q$.

#### **Definition 33**

(*Lookahead*) A transition rule contains lookahead if a variable occurs at the left-hand side of a premise and at the right-hand side of a premise of this rule.

#### **Definition 34**

(*Patience rule*) A patience rule for the ith argument of a function symbol *f* is a panth rule of the form

$$\begin{aligned} \frac{x_i\xrightarrow {\tau }y}{f(x_1,\ldots ,x_{ar(f)})\xrightarrow {\tau }f(x_1,\ldots ,x_{i-1},y,x_{i+1},\ldots ,x_{ar(f)})}. \end{aligned}$$

#### **Definition 35**

(*RBB cool format*) A TSS *T* is in RBB cool format if the following requirements are fulfilled. (1) *T* consists of panth rules that do not contain lookahead. (2) Suppose a function symbol *f* occurs at the right-hand side the conclusion of some transition rule in *T*. Let $\rho \in T$ be a non-patience rule with source $f(x_1,\ldots ,x_{ar(f)})$. Then for $i\in \{1,\ldots ,ar(f)\}, x_i$ occurs in no more than one premise of $\rho$, where this premise is of the form $x_iP$ or $x_i\xrightarrow {a}y$ with $a\ne \tau$. Moreover, if there is such a premise in $\rho$, then there is a patience rule for the i-th argument of *f* in *T*.

#### **Theorem 5**

*If a TSS is positive after reduction and in RBB cool format, then the rooted branching bisimulation equivalence that it induces is a congruence.*

#### **Definition 36**

(*Conservative extension*) Let $T_0$ and $T_1$ be TSSs over signatures $\varSigma _0$ and $\varSigma _1$, respectively. The TSS $T_0\oplus T_1$ is a conservative extension of $T_0$ if the LTSs generated by $T_0$ and $T_0\oplus T_1$ contain exactly the same transitions $t\xrightarrow {a}t'$ and *tP* with $t\in {\mathcal {T}}(\varSigma _0)$.

#### **Definition 37**

(*Source-dependency*) The source-dependent variables in a transition rule of $\rho$ are defined inductively as follows: (1) all variables in the source of $\rho$ are source-dependent; (2) if $t\xrightarrow {a}t'$ is a premise of $\rho$ and all variables in *t* are source-dependent, then all variables in $t'$ are source-dependent. A transition rule is source-dependent if all its variables are. A TSS is source-dependent if all its rules are.

#### **Definition 38**

(*Freshness*) Let $T_0$ and $T_1$ be TSSs over signatures $\varSigma _0$ and $\varSigma _1$, respectively. A term in ${\mathbb {T}}(T_0\oplus T_1)$ is said to be fresh if it contains a function symbol from $\varSigma _1{\setminus}\varSigma _0$. Similarly, a transition label or predicate symbol in $T_1$ is fresh if it does not occur in $T_0$.

#### **Theorem 6**

*Let*$T_0$*and*$T_1$*be TSSs over signatures*$\varSigma _0$*and*$\varSigma _1$,*respectively, where*$T_0$*and*$T_0\oplus T_1$*are positive after reduction. Under the following conditions,*$T_0\oplus T_1$*is a conservative extension of*$T_0$. *(1)*$T_0$*is source-dependent. (2) For each*$\rho \in T_1$, *either the source of*$\rho$*is fresh, or*$\rho$*has a premise of the form*$t\xrightarrow {a}t'$*ortP*, *where*$t\in {\mathbb {T}}(\varSigma _0)$, *all variables intoccur in the source of*$\rho$*and*$t', a$*orPis fresh*.

### Process algebra: ACP

ACP (Fokkink 2007) is a kind of process algebra which focuses on the specification and manipulation of process terms by use of a collection of operator symbols. In ACP, there are several kind of operator symbols, such as basic operators to build finite processes (called BPA), communication operators to express concurrency (called PAP), deadlock constants and encapsulation enable us to force actions into communications (called ACP), liner recursion to capture infinite behaviors (called ACP with linear recursion), the special constant silent step and abstraction operator (called $ACP_{\tau }$ with guarded linear recursion) allows us to abstract away from internal computations.

Bisimulation or rooted branching bisimulation based structural operational semantics is used to formally provide each process term used the above operators and constants with a process graph. The axiomatization of ACP (according the above classification of ACP, the axiomatizations are ${\mathcal {E}}_{\text {BPA}}, {\mathcal {E}}_{\text {PAP}}, {\mathcal {E}}_{\text {ACP}}, {\mathcal {E}}_{\text {ACP}}$ + RDP (Recursive Definition Principle) + RSP (Recursive Specification Principle), ${\mathcal {E}}_{\text {ACP}_\tau }$ + RDP + RSP + CFAR (Cluster Fair Abstraction Rule) respectively) imposes an equation logic on process terms, so two process terms can be equated if and only if their process graphs are equivalent under the semantic model.

ACP can be used to formally reason about the behaviors, such as processes executed sequentially and concurrently by use of its basic operator, communication mechanism, and recursion, desired external behaviors by its abstraction mechanism, and so on.

ACP is organized by modules and can be extended with fresh operators to express more properties of the specification for system behaviors. These extensions are required both the equational logic and the structural operational semantics to be extended. Then the extension can use the whole outcomes of ACP, such as its concurrency, recursion, abstraction, etc.

## BRPA: basic reversible process algebra

In the following, the variables $x,x',y,y',z,z'$ range over the collection of process terms, the variables $\upsilon ,\omega$ range over the set *A* of atomic actions, $a,b\in A, s,s',t,t'$ are closed items, $\tau$ is the special constant silent step, $\delta$ is the special constant deadlock. We define a kind of special action constant $a[m]\in A \times {\mathcal {K}}$ where ${\mathcal {K}}\subseteq {\mathbb {N}}$, called the histories of an action *a*, denoted by $a[m],a[n],\ldots$ where $m,n\in {\mathcal {K}}$. Let $A=A\cup \{A\times {\mathcal {K}}\}$.

BRPA includes three kind of operators: the execution of atomic action *a*, the choice composition operator + and the sequential composition operator $\cdot$. Each finite process can be represented by a closed term that is built from the set *A* of atomic actions or histories of an atomic action, the choice composition operator +, and the sequential composition operator $\cdot$. The collection of all basic process terms is called Basic Reversible Process Algebra (BRPA), which is abbreviated to BRPA.

### Transition rules of BRPA

We give the forward transition rules under transition system specification (TSS) for BRPA as follows.

$$\begin{aligned}&\frac{}{\upsilon \xrightarrow {\upsilon }\upsilon [m]}\\&\frac{x\xrightarrow {\upsilon }\upsilon [m] \quad \upsilon \notin y}{x+y\xrightarrow {\upsilon }\upsilon [m]+y} \quad \frac{x\xrightarrow {\upsilon }x' \quad \upsilon \notin y}{x+y\xrightarrow {\upsilon }x'+y} \quad \frac{y\xrightarrow {\upsilon }\upsilon [m] \quad \upsilon \notin x}{x+y\xrightarrow {\upsilon }x+\upsilon [m]} \quad \frac{y\xrightarrow {\upsilon }y'\quad \upsilon \notin x}{x+y\xrightarrow {\upsilon }x+y'}\\&\frac{x\xrightarrow {\upsilon }\upsilon [m]\quad y\xrightarrow {\upsilon }\upsilon [m]}{x+y\xrightarrow {\upsilon }\upsilon [m]} \quad \frac{x\xrightarrow {\upsilon }x'\quad y\xrightarrow {\upsilon }\upsilon [m]}{x+y\xrightarrow {\upsilon }x'+\upsilon [m]} \quad \frac{x\xrightarrow {\upsilon }\upsilon [m]\quad y\xrightarrow {\upsilon }y'}{x+y\xrightarrow {\upsilon }\upsilon [m]+y'} \quad \frac{x\xrightarrow {\upsilon }x'\quad y\xrightarrow {\upsilon }y'}{x+y\xrightarrow {\upsilon }x'+y'}\\&\frac{x\xrightarrow {\upsilon }\upsilon [m]}{x\cdot y\xrightarrow {\upsilon }\upsilon [m]\cdot y} \quad \frac{x\xrightarrow {\upsilon }x'}{x\cdot y\xrightarrow {\upsilon }x'\cdot y}\\&\frac{y\xrightarrow {\omega }\omega [n]}{x\cdot y\xrightarrow {\omega }x\cdot \omega [n]}, \hbox { x is forward executed successfully.}\\&\frac{y\xrightarrow {\omega }y'}{x\cdot y\xrightarrow {\omega }x\cdot y'}, \hbox { x is forward executed successfully.} \end{aligned}$$

- The first transition rule says that each atomic action $\upsilon$ can execute successfully, and leads to a history $\upsilon [m]$. The forward transition rule $\frac{}{\upsilon \xrightarrow {\upsilon }\upsilon [m]}$ implies a successful forward execution.
- The next four transition rules say that $s+t$ can execute only one branch, that is, it can execute either *s* or *t*, but the other branch remains.
- The next four transition rules say that $s+t$ can execute both branches, only by executing the same atomic actions. When one branch *s* or *t* is forward executed successfully, we define $s+t$ is forward executed successfully.
- The last four transition rules say that $s\cdot t$ can execute sequentially, that is, it executes *s* in the first and leads to a successful history, after successful execution of *s*, then execution of *t* follows. When both *s* and *t* are forward executed successfully, we define $s\cdot t$ is forward executed successfully.

We give the reverse transition rules under transition system specification (TSS) for BRPA as follows.

$$\begin{aligned}&\frac{}{\upsilon [m]\mathop {\twoheadrightarrow }\limits ^{\upsilon [m]}\upsilon }\\&\frac{x\mathop {\twoheadrightarrow }\limits ^{\upsilon [m]}\upsilon \quad \upsilon [m]\notin y}{x+y\mathop {\twoheadrightarrow }\limits ^{\upsilon [m]}\upsilon +y} \quad \frac{x\mathop {\twoheadrightarrow }\limits ^{\upsilon [m]}x' \quad \upsilon [m]\notin y}{x+y\mathop {\twoheadrightarrow }\limits ^{\upsilon [m]}x'+y} \quad \frac{y\mathop {\twoheadrightarrow }\limits ^{\upsilon [m]}\upsilon \quad \upsilon [m]\notin x}{x+y\mathop {\twoheadrightarrow }\limits ^{\upsilon [m]}x+\upsilon } \quad \frac{y\mathop {\twoheadrightarrow }\limits ^{\upsilon [m]}y' \quad \upsilon [m]\notin x}{x+y\mathop {\twoheadrightarrow }\limits ^{\upsilon [m]}x+y'}\\&\frac{x\mathop {\twoheadrightarrow }\limits ^{\upsilon [m]}\upsilon \quad y\mathop {\twoheadrightarrow }\limits ^{\upsilon [m]}\upsilon }{x+y\mathop {\twoheadrightarrow }\limits ^{\upsilon [m]}\upsilon } \quad \frac{x\mathop {\twoheadrightarrow }\limits ^{\upsilon [m]}x'\quad y\mathop {\twoheadrightarrow }\limits ^{\upsilon [m]}\upsilon }{x+y\mathop {\twoheadrightarrow }\limits ^{\upsilon [m]}x'+\upsilon } \quad \frac{x\mathop {\twoheadrightarrow }\limits ^{\upsilon [m]}\upsilon \quad y\mathop {\twoheadrightarrow }\limits ^{\upsilon [m]}y'}{x+y\mathop {\twoheadrightarrow }\limits ^{\upsilon [m]}\upsilon +y'} \quad \frac{x\mathop {\twoheadrightarrow }\limits ^{\upsilon [m]}x'\quad y\mathop {\twoheadrightarrow }\limits ^{\upsilon [m]}y'}{x+y\mathop {\twoheadrightarrow }\limits ^{\upsilon [m]}x'+y'}\\&\frac{x\mathop {\twoheadrightarrow }\limits ^{\upsilon [m]}\upsilon }{x\cdot y\mathop {\twoheadrightarrow }\limits ^{\upsilon [m]}\upsilon \cdot y} \quad \frac{x\mathop {\twoheadrightarrow }\limits ^{\upsilon [m]}x'}{x\cdot y\mathop {\twoheadrightarrow }\limits ^{\upsilon [m]}x'\cdot y} \\&\frac{y\mathop {\twoheadrightarrow }\limits ^{\omega [n]}\omega }{x\cdot y\mathop {\twoheadrightarrow }\limits ^{\omega [n]}x\cdot \omega }, \hbox { x is forward executed successfully }. \\&\frac{y\mathop {\twoheadrightarrow }\limits ^{\omega [n]}y'}{x\cdot y\mathop {\twoheadrightarrow }\limits ^{\omega [n]}x\cdot y'},\hbox { x is forward executed successfully }. \end{aligned}$$

- The first transition rule says that each history of an atomic action $\upsilon [m]$ can reverse successfully, and leads to an atomic action $\upsilon$. Similarly, the reverse transition rule $\frac{}{\upsilon [m]\mathop {\twoheadrightarrow }\limits ^{\upsilon [m]}\upsilon }$ implies a successful reverse.
- The next four transition rules say that $s+t$ can reverse only one branch, that is, it can reverse either *s* or *t*, but the other branch remains.
- The next four transition rules say that $s+t$ can reverse both branches, only by executing the same histories of atomic actions. When one branch *s* or *t* is reversed successfully, we define $s+t$ is reversed successfully.
- The last four transition rules say that $s\cdot t$ can reverse sequentially, that is, it reverses *t* in the first and leads to a successful atomic action, after successful reverse of *t*, then reverse of *s* follows. When both *s* and *t* are reversed successfully, we define $s\cdot t$ is reversed successfully.

### Axiomatization for BRPA

We design an axiomatization ${\mathcal {E}}_{\text {BRPA}}$ for BRPA modulo FR bisimulation equivalence as Table 1 shows.

**Table 1:** Axioms for BRPA

No.	Axiom
RA1	$x + y = y + x$
RA2	$x + x = x$
RA3	$(x + y) + z = x + (y + z)$
RA4	$x \cdot (y + z) = x\cdot y + x\cdot z$
RA5	$(x\cdot y)\cdot z = x\cdot (y\cdot z)$

The following conclusions can be obtained.

#### **Theorem 7**

*FR bisimulation equivalence is a congruence with respect to BRPA.*

#### *Proof*

The forward and reverse TSSs are all in panth format, so FR bisimulation equivalence that they induce is a congruence. $\square$

#### **Theorem 8**

${\mathcal {E}}_{\text {BRPA}}$*is sound for BRPA modulo FR bisimulation equivalence*.

#### *Proof*

Since FR bisimulation is both an equivalence and a congruence for BRPA, only the soundness of the first clause in the definition of the relation = is needed to be checked. That is, if $s=t$ is an axiom in ${\mathcal {E}}_{\text {BRPA}}$ and $\sigma$ a closed substitution that maps the variable in *s* and *t* to basic reversible process terms, then we need to check that $\sigma (s)\underline{\leftrightarrow }^{fr}\sigma (t)$.

We only provide some intuition for the soundness of the axioms in Table 1.

- RA1 (commutativity of +) says that $s+t$ and $t+s$ are all execution branches and are equal modulo FR bisimulation.
- RA2 (idempotency of +) is used to eliminate redundant branches.
- RA3 (associativity of +) says that $(s+t)+u$ and $s+(t+u)$ are all execution branches of *s*, *t*, *u*.
- RA4 (left distributivity of $\cdot$) says that both $s \cdot (t+u)$ and $s\cdot t + s\cdot u$ represent the same execution branches. It must be pointed out that the right distributivity of $\cdot$ does not hold modulo FR bisimulation. For example, $(a+b)\cdot c\xrightarrow {a}(a[m]+b)\cdot c\xrightarrow {c}(a[m]+b)\cdot c[n]\mathop {\twoheadrightarrow }\limits ^{c[n]}(a[m]+b)\cdot c\mathop {\twoheadrightarrow}{a[m]}(a+b)\cdot c;$ while $a\cdot c + b\cdot c\xrightarrow {a}a[m]\cdot c+b\cdot c\mathop {\nrightarrow }\limits ^{c}$.
- RA5 (associativity of $\cdot$) says that both $(s\cdot t)\cdot u$ and $s\cdot (t\cdot u)$ represent forward execution of *s* followed by *t* followed by *u*, or, reverse execution of *u* followed by *t* followed by *s*.

These intuitions can be made rigorous by means of explicit FR bisimulation relations between the left- and right-hand sides of closed instantiations of the axioms in Table 1. Hence, all such instantiations are sound modulo FR bisimulation equivalence. $\square$

#### **Theorem 9**

${\mathcal {E}}_{\text {BRPA}}$*is complete for BRPA modulo FR bisimulation equivalence*.

#### *Proof*

We refer to Fokkink (2007) for the completeness proof of ${\mathcal {E}}_{\text {BPA}}$.

To prove that ${\mathcal {E}}_{\text {BRPA}}$ is complete for BRPA modulo FR bisilumation equivalence, it means that $s\underline{\leftrightarrow }^{fr} t$ implies $s=t$.

We consider basic reversible process terms modulo associativity and commutativity (*AC*) of the + (RA1,RA2), and this equivalence relation is denoted by $=_{AC}$. A basic reversible process term *s* then represents the collection of basic reversible process term *t* such that $s=_{AC} t$. Each equivalence class *s* modulo *AC* of the + can be represented in the form $s_1+\cdots +s_k$ with each $s_i$ either an atomic action or of the form $t_1\cdot t_2$. We refer to the subterms $s_1,\ldots ,s_k$ as the summands of *s*.

Then RA3-RA5 are turned into rewrite rules from left to right:

$$\begin{aligned}&x + x \rightarrow x\\&x \cdot (y + z) \rightarrow x\cdot y + x\cdot z\\&(x\cdot y)\cdot z \rightarrow x\cdot (y\cdot z). \end{aligned}$$

Then these rewrite rules are applied to basic reversible process terms modulo *AC* of the +.

We let the weight functions

$$\begin{aligned}&weight(\upsilon )\triangleq 2\\&weight(\upsilon [m])\triangleq 2\\&weight(s+t)\triangleq weight(s)+weight(t)\\&weight(s\cdot t)\triangleq weight(s)\cdot weight(t)^2. \end{aligned}$$

We can see that the TRS is terminating modulo *AC* of the +.

Next, we prove that normal forms *n* and $n'$ with $n\underline{\leftrightarrow }^{fr} n'$ implies $n=_{AC} n'$. The proof is based on induction with respect to the sizes of *n* and $n'$. Let $n\underline{\leftrightarrow }^{fr} n'$.

- Consider a summand *a* of *n*. Then $n\xrightarrow {a}a[m]+u$, so $n\underline{\leftrightarrow }^{fr} n'$ implies $n'\xrightarrow {a}a[m]+u$, meaning that $n'$ also contains the summand *a*.
- Consider a summand *a*[*m*] of *n*. Then $n\mathop {\twoheadrightarrow}{a[m]}a+u$, so $n\underline{\leftrightarrow }^{fr} n'$ implies $n'\mathop {\twoheadrightarrow}{a[m]}a+u$, meaning that $n'$ also contains the summand *a*[*m*].
- Consider a summand $a_1\ldots a_i\ldots a_k$ of *n*. Then $n\xrightarrow {a_1}\cdots \xrightarrow {a_i}\cdots \xrightarrow {a_k} a_1[m_1]\ldots a_i[m_i]\ldots a_k[m_k]+u$, so $n\underline{\leftrightarrow }^{fr} n'$ implies $n'\xrightarrow {a_1}\cdots \xrightarrow {a_i}\cdots \xrightarrow {a_k} a_1[m_1]\ldots a_i[m_i]\ldots a_k[m_k]+u$, meaning that $n'$ also contains the summand $a_1\ldots a_i\ldots a_k$.
- Consider a summand $a_1[m_1]\ldots a_i[m_i]\ldots a_k[m_k]$ of *n*. Then $n\mathop {\twoheadrightarrow }\limits ^{a_k[m_k]}\cdots \mathop {\twoheadrightarrow }\limits ^{a_i[m_i]}\cdots \mathop {\twoheadrightarrow }\limits ^{a_1[m_1]}a_1\ldots a_i\ldots a_k+u$, so $n\underline{\leftrightarrow }^{fr} n'$ implies $n'\mathop {\twoheadrightarrow }\limits ^{a_k[m_k]}\cdots \mathop {\twoheadrightarrow }\limits ^{a_i[m_i]}\cdots \mathop {\twoheadrightarrow }\limits ^{a_1[m_1]}a_1\ldots a_i\ldots a_k+u$, meaning that $n'$ also contains the summand $a_1[m_1]\ldots a_i[m_i]\ldots a_k[m_k]$.

Hence, each summand of *n* is also a summand of $n'$. Vice versa, each summand of $n'$ is also a summand of *n*. In other words, $n=_{AC} n'$.

Finally, let the basic reversible process terms *s* and *t* be FR bisimilar. The TRS is terminating modulo *AC* of the +, so it reduces *s* and *t* to normal forms *n* and $n'$, respectively. Since the rewrite rules and equivalence modulo *AC* of the + can be derived from the axioms, $s=n$ and $t=n'$. Soundness of the axioms then yields $s\underline{\leftrightarrow }^{fr} n$ and $t\underline{\leftrightarrow }^{fr} n'$, so $n\underline{\leftrightarrow }^{fr} s\underline{\leftrightarrow }^{fr} t\underline{\leftrightarrow }^{fr} n'$. We showed that $n\underline{\leftrightarrow }^{fr} n'$ implies $n=_{AC}n'$. Hence, $s=n=_{AC} n'=t$. $\square$

## ARCP: algebra of reversible communicating processes

It is well known that process algebra captures parallelism and concurrency by means of the so-called interleaving pattern in contrast to the so-called true concurrency. ACP uses left merge and communication merge to bridge the gap between the parallel semantics, and sequential semantics. But in reversible computation, Milner’s expansion law modeled by left merge does not hold any more, as pointed out in Phillips (2007). $a\parallel b \ne a\cdot b + b\cdot a$, because $a\parallel b\xrightarrow {a}a[m]\parallel b\xrightarrow {b}a[m]\parallel b[n]$ and $a\cdot b + b\cdot a\mathop {\nrightarrow }\limits ^{a}$. That is, the left merge to capture the asynchronous concurrency in an interleaving fashion will be instead by a real static parallel fashion and the parallel branches cannot be merged. But, the communication merge used to capture synchrony will be retained.

### Static parallelism and communication merge

We use a parallel operator $\parallel$ to represent the whole parallelism semantics, a static parallel ${\text{operator}}\,|$ to represent the real parallelism semantics, and a communication merge $\between$ to represent the synchronisation. We call BRPA extended with the whole parallel operator $\parallel$, the static parallel ${\text{operator}}\,|$ and the communication merge operator $\between$ Reversible Process Algebra with Parallelism, which is abbreviated to RPAP.

#### Transition rules of RPAP

We give the forward transition rules under transition system specification (TSS) for the static parallel ${\text{operator}}\,|$ as follows.

$$\begin{aligned}&\frac{x\xrightarrow {\upsilon }\upsilon [m]}{x\mid y\xrightarrow {\upsilon }\upsilon [m]\mid y} \quad \frac{x\xrightarrow {\upsilon }x'}{x\mid y\xrightarrow {\upsilon }x'\mid y} \quad \frac{y\xrightarrow {\upsilon }\upsilon [m]}{x\mid y\xrightarrow {\upsilon }x\mid \upsilon [m]} \quad \frac{y\xrightarrow {\upsilon }y'}{x\mid y\xrightarrow {\upsilon }x\mid y'}.\\&\frac{x\xrightarrow {\upsilon }\upsilon [m]\quad y\xrightarrow {\upsilon }\upsilon [m]}{x\mid y\xrightarrow {\upsilon }\upsilon [m]} \quad \frac{x\xrightarrow {\upsilon }x'\quad y\xrightarrow {\upsilon }\upsilon [m]}{x\mid y\xrightarrow {\upsilon }x'\mid \upsilon [m]} \quad \frac{x\xrightarrow {\upsilon }\upsilon [m]\quad y\xrightarrow {\upsilon }y'}{x\mid y\xrightarrow {\upsilon }\upsilon [m]\mid y'} \quad \frac{x\xrightarrow {\upsilon }x'\quad y\xrightarrow {\upsilon }y'}{x\mid y\xrightarrow {\upsilon }x'\parallel y'}.\\ \end{aligned}$$

The above eight transition rules are forward transition rules for the static parallel ${\text{operator}}\,|$ and state that $s\mid t$ can execute in a real parallel pattern. When both *s* and *t* are forward executed successfully, we define $s\mid t$ is forward executed successfully.

$$\begin{aligned}&\frac{x\mathop {\twoheadrightarrow }\limits ^{\upsilon [m]}\upsilon }{x\mid y\mathop {\twoheadrightarrow }\limits ^{\upsilon [m]}\upsilon \mid y} \quad \frac{x\mathop {\twoheadrightarrow }\limits ^{\upsilon [m]}x'}{x\mid y\mathop {\twoheadrightarrow }\limits ^{\upsilon [m]}x'\mid y} \quad \frac{y\mathop {\twoheadrightarrow }\limits ^{\upsilon [m]}\upsilon }{x\mid y\mathop {\twoheadrightarrow }\limits ^{\upsilon [m]}x\mid \upsilon } \quad \frac{y\mathop {\twoheadrightarrow }\limits ^{\upsilon [m]}y'}{x\mid y\mathop {\twoheadrightarrow }\limits ^{\upsilon [m]}x\mid y'}.\\&\frac{x\mathop {\twoheadrightarrow }\limits ^{\upsilon [m]}\upsilon \quad y\mathop {\twoheadrightarrow }\limits ^{\upsilon [m]}\upsilon }{x\mid y\mathop {\twoheadrightarrow }\limits ^{\upsilon [m]}\upsilon } \quad \frac{x\mathop {\twoheadrightarrow }\limits ^{\upsilon [m]}x'\quad y\mathop {\twoheadrightarrow }\limits ^{\upsilon [m]}\upsilon }{x\mid y\mathop {\twoheadrightarrow }\limits ^{\upsilon [m]}x'\mid \upsilon } \quad \frac{x\mathop {\twoheadrightarrow }\limits ^{\upsilon [m]}\upsilon \quad y\mathop {\twoheadrightarrow }\limits ^{\upsilon [m]}y'}{x\mid y\mathop {\twoheadrightarrow }\limits ^{\upsilon [m]}\upsilon \mid y'} \quad \frac{x\mathop {\twoheadrightarrow }\limits ^{\upsilon [m]}x'\quad y\mathop {\twoheadrightarrow }\limits ^{\upsilon [m]}y'}{x\mid y\mathop {\twoheadrightarrow }\limits ^{\upsilon [m]}x'\parallel y'}. \end{aligned}$$

The above eight transition rules are reverse transition rules for the static parallel ${\text{operator}}\,|$ and say that $s\mid t$ can reverse in a real parallel pattern. When both *s* and *t* are reversed successfully, we define $s\mid t$ is reversed successfully.

The forward transition rules under TSS for communication merge are as follows and say that the communication can be merged. Where a communication function $\gamma :A\times A\rightarrow A$ is defined.

$$\begin{aligned}&\frac{x\xrightarrow {\upsilon }\upsilon [m]\quad y\xrightarrow {\omega }\omega [m]}{x\between y\xrightarrow {\gamma (\upsilon ,\omega )}\gamma (\upsilon ,\omega )[m]} \quad \frac{x\xrightarrow {\upsilon }\upsilon [m]\quad y\xrightarrow {\omega }y'}{x\between y\xrightarrow {\gamma (\upsilon ,\omega )}\gamma (\upsilon ,\omega )[m]\cdot y'}\\&\frac{x\xrightarrow {\upsilon }x'\quad y\xrightarrow {\omega }\omega [m]}{x\between y\xrightarrow {\gamma (\upsilon ,\omega )}\gamma (\upsilon ,\omega )[m]\cdot x'} \quad \frac{x\xrightarrow {\upsilon }x'\quad y\xrightarrow {\omega }y'}{x\between y\xrightarrow {\gamma (\upsilon ,\omega )}\gamma (\upsilon ,\omega )[m]\cdot x'\parallel y'}. \end{aligned}$$

The reverse transition rules under TSS for communication merge are as follows and say that the communication can be merged.

$$\begin{aligned}&\frac{x\mathop {\twoheadrightarrow }\limits ^{\upsilon [m]}\upsilon \quad y\mathop {\twoheadrightarrow }\limits ^{\omega [m]}\omega }{x\between y\mathop {\twoheadrightarrow }\limits ^{\gamma (\upsilon ,\omega )[m]}\gamma (\upsilon ,\omega )} \quad \frac{x\mathop {\twoheadrightarrow }\limits ^{\upsilon [m]}\upsilon \quad y\mathop {\twoheadrightarrow }\limits ^{\omega [m]}y'}{x\between y\mathop {\twoheadrightarrow }\limits ^{\gamma (\upsilon ,\omega )[m]}\gamma (\upsilon ,\omega )\cdot y'}\\&\frac{x\mathop {\twoheadrightarrow }\limits ^{\upsilon [m]}x'\quad y\mathop {\twoheadrightarrow }\limits ^{\omega [m]}\omega }{x\between y\mathop {\twoheadrightarrow }\limits ^{\gamma (\upsilon ,\omega )[m]}\gamma (\upsilon ,\omega )\cdot x'} \quad \frac{x\mathop {\twoheadrightarrow }\limits ^{\upsilon [m]}x'\quad y\mathop {\twoheadrightarrow }\limits ^{\omega [m]}y'}{x\between y\mathop {\twoheadrightarrow }\limits ^{\gamma (\upsilon ,\omega )[m]}\gamma (\upsilon ,\omega )\cdot x'\parallel y'}. \end{aligned}$$

##### **Theorem 10**

*RPAP is a conservative extension of BRPA.*

##### *Proof*

Since the TSS of BRPA is source-dependent, and the transition rules for the static parallel ${\text{operator}}\,|$, communication merge $\between$ contain only a fresh operator in their source, so the TSS of RPAP is a conservative extension of that of BRPA. That means that RPAP is a conservative extension of BRPA. $\square$

##### **Theorem 11**

*FR bisimulation equivalence is a congruence with respect to RPAP.*

##### *Proof*

The TSSs for RPAP and BRPA are all in panth format, so FR bisimulation equivalence that they induce is a congruence. $\square$

#### Axiomatization for RPAP

We design an axiomatization for RPAP illustrated in Table 2.

**Table 2:** Axioms for RPAP

No.	Axiom
RP1	$x\parallel y = x\mid y + x\between y$
RP2	$x\mid x = x$
RP3	$(x\mid y)\mid z = x\mid (y\mid z)$
RP4	$x \mid (y + z) = x\mid y + x\mid z$
RP5	$(x + y) \mid z = x\mid z + y\mid z$
RP6	$x \cdot (y \mid z) = x\cdot y \mid x\cdot z$
RP7	$(x \mid y) \cdot z = x\cdot z \mid y\cdot z$
RC8	$\upsilon \between \omega =\gamma (\upsilon ,\omega )$
RC9	$\upsilon [m]\between \omega [m]=\gamma (\upsilon ,\omega )[m]$
RC10	$\upsilon \between (\omega \cdot y) = \gamma (\upsilon ,\omega )\cdot y$
RC11	$\upsilon [m]\between (\omega [m]\cdot y) = \gamma (\upsilon ,\omega )[m]\cdot y$
RC12	$(\upsilon \cdot x)\between \omega = \gamma (\upsilon ,\omega )\cdot x$
RC13	$(\upsilon [m]\cdot x)\between \omega [m] = \gamma (\upsilon ,\omega )[m]\cdot x$
RC14	$(\upsilon \cdot x)\between (\omega \cdot y) = \gamma (\upsilon ,\omega )\cdot (x\parallel y)$
RC15	$(\upsilon [m]\cdot x)\between (\omega [m]\cdot y) = \gamma (\upsilon ,\omega )[m]\cdot (x\parallel y)$
RC16	$(x + y)\between z = x\between z + y\between z$
RC17	$x\between (y+z) = x\between y + x\between z$

Then, we can obtain the soundness and completeness theorems as follows.

##### **Theorem 12**

${\mathcal {E}}_{\text {RPAP}}$*is sound for RPAP modulo FR bisimulation equivalence.*

##### *Proof*

Since FR bisimulation is both an equivalence and a congruence for RPAP, only the soundness of the first clause in the definition of the relation = is needed to be checked. That is, if $s=t$ is an axiom in ${\mathcal {E}}_{\text {RPAP}}$ and $\sigma$ a closed substitution that maps the variable in *s* and *t* to reversible process terms, then we need to check that $\sigma (s)\underline{\leftrightarrow }^{fr}\sigma (t)$.

We only provide some intuition for the soundness of the axioms in Table 2.

- RP1 says that $s\parallel t$ is a real static parallel or is a communication of initial transitions from *s* and *t*.
- RP2 says that $s\mid s$ can eliminate redundant parallel branches to *s*.
- RP3-RP7 say that the static parallel operator satisfies associativity, left distributivity and right distributivity to + and $\cdot$.
- RC8-RC15 are the defining axioms for the communication merge, which say that $s\between t$ makes as initial transition a communication of initial transitions from *s* and *t*.
- RC16-RC17 say that the communication merge $\between$ satisfies both left distributivity and right distributivity.

These intuitions can be made rigorous by means of explicit FR bisimulation relations between the left- and right-hand sides of closed instantiations of the axioms in Table 2. Hence, all such instantiations are sound modulo FR bisimulation equivalence. $\square$

##### **Theorem 13**

${\mathcal {E}}_{\text {RPAP}}$*is complete for RPAP modulo FR bisimulation equivalence.*

##### *Proof*

To prove that ${\mathcal {E}}_{\text {RPAP}}$ is complete for RPAP modulo FR bisilumation equivalence, it means that $s\underline{\leftrightarrow }^{fr} t$ implies $s=t$.

(1) We consider the introduction to the static ${\text{parallel}}\,|$.

We consider reversible process terms contains +, $\cdot , \mid$ modulo associativity and commutativity (*AC*) of the + (RA1,RA2), and this equivalence relation is denoted by $=_{AC}$. A reversible process term *s* then represents the collection of reversible process term *t* contains $+, \cdot$, ${\text{and}}\,|$ such that $s =_{AC} t$. Each equivalence class *s* modulo *AC* of the + can be represented in the form $s_{11}\mid \ldots \mid s_{1l}+\cdots +s_{k1}\mid \ldots \mid s_{km}$ with each $s_{ij}$ either an atomic action or of the form $t_1\cdot t_2$. We refer to the subterms $s_{ij}$ and $s_{ij}\mid s_{i,j+1}$ are the summands of *s*.

Then RP2-RP7 are turned into rewrite rules from left to right:

$$\begin{aligned}&x\mid x \rightarrow x\\&(x\mid y)\mid z \rightarrow x\mid (y\mid z)\\&x \mid (y + z) \rightarrow x\mid y + x\mid z\\&(x + y)\mid z \rightarrow x\mid z + y \mid z\\&x \cdot (y \mid z) \rightarrow x\cdot y \mid x\cdot z\\&(x \mid y) \cdot z \rightarrow x\cdot z \mid y\cdot z. \end{aligned}$$

Then these rewrite rules are applied to the above reversible process terms modulo *AC* of the +.

We let the weight function

$$\begin{aligned}&weight(\upsilon )\triangleq 2\\&weight(\upsilon [m])\triangleq 2\\&weight(s+t)\triangleq weight(s)+weight(t)\\&weight(s\cdot t)\triangleq weight(s)^3\cdot weight(t)^3\\&weight(s\mid t)\triangleq weight(s)^2\cdot weight(t)^2. \end{aligned}$$

We can see that the TRS is terminating modulo *AC* of the +.

Next, we prove that normal forms *n* and $n'$ with $n\underline{\leftrightarrow }^{fr} n'$ implies $n=_{AC} n'$. The proof is based on induction with respect to the sizes of *n* and $n'$. Let $n\underline{\leftrightarrow }^{fr} n'$.

- Consider a summand *a* of *n*. Then $n\xrightarrow {a}a[m]+u$, so $n\underline{\leftrightarrow }^{fr} n'$ implies $n'\xrightarrow {a}a[m]+u$, meaning that $n'$ also contains the summand *a*.
- Consider a summand *a*[*m*] of *n*. Then $n\mathop {\twoheadrightarrow}{a[m]}a+u$, so $n\underline{\leftrightarrow }^{fr} n'$ implies $n'\mathop {\twoheadrightarrow}{a[m]}a+u$, meaning that $n'$ also contains the summand *a*[*m*].
- Consider a summand $a_1\ldots a_i\ldots a_k$ of *n*. Then $n\xrightarrow {a_1}\cdots \xrightarrow {a_i}\cdots \xrightarrow {a_k} a_1[m_1]\ldots a_i[m_i]\ldots a_k[m_k]+u$, so $n\underline{\leftrightarrow }^{fr} n'$ implies $n'\xrightarrow {a_1}\cdots \xrightarrow {a_i}\cdots \xrightarrow {a_k} a_1[m_1]\ldots a_i[m_i]\ldots a_k[m_k]+u$, meaning that $n'$ also contains the summand $a_1\ldots a_i\ldots a_k$.
- Consider a summand $a_1[m_1]\ldots a_i[m_i]\ldots a_k[m_k]$ of *n*. Then $n\mathop {\twoheadrightarrow }\limits ^{a_k[m_k]}\ldots \mathop {\twoheadrightarrow }\limits ^{a_i[m_i]}\ldots \mathop {\twoheadrightarrow }\limits ^{a_1[m_1]}a_1\ldots a_i\ldots a_k+u$, so $n\underline{\leftrightarrow }^{fr} n'$ implies $n'\mathop {\twoheadrightarrow }\limits ^{a_k[m_k]}\ldots \mathop {\twoheadrightarrow }\limits ^{a_i[m_i]}\ldots \mathop {\twoheadrightarrow }\limits ^{a_1[m_1]}a_1\ldots a_i\ldots a_k+u$, meaning that $n'$ also contains the summand $a_1[m_1]\ldots a_i[m_i]\ldots a_k[m_k]$.
- Consider a summand $a\mid b$ of *n*. Then $n\xrightarrow {a}a[m]\mid b+u\xrightarrow {b}a[m]\mid b[k]+u$, or $n\xrightarrow {b}a\mid b[k]+u\xrightarrow {a}a[m]\mid b[k]+u$, so $n\underline{\leftrightarrow }^{fr} n'$ implies $n'\xrightarrow {a}a[m]\mid b+u\xrightarrow {b}a[m]\mid b[k]+u$, or $n'\xrightarrow {b}a\mid b[k]+u\xrightarrow {a}a[m]\mid b[k]+u$, meaning that $n'$ also contains the summand $a\mid b$.
- Consider a summand $a[m]\mid b[k]$ of *n*. Then $n\mathop {\twoheadrightarrow}{a[m]}a\mid b[k]+u\mathop {\twoheadrightarrow }\limits ^{b[k]}a\mid b+u$, or $n\mathop {\twoheadrightarrow }\limits ^{b[k]}a[m]\mid b+u\mathop {\twoheadrightarrow}{a[m]}a\mid b+u$, so $n\underline{\leftrightarrow }^{fr} n'$ implies $n'\mathop {\twoheadrightarrow}{a[m]}a\mid b[k]+u\mathop {\twoheadrightarrow }\limits ^{b[k]}a\mid b+u$, or $n'\mathop {\twoheadrightarrow }\limits ^{b[k]}a[m]\mid b+u\mathop {\twoheadrightarrow}{a[m]}a\mid b+u$, meaning that $n'$ also contains the summand $a[m]\mid b[k]$.
- The summands $as\mid bt$ and $a[m]s\mid b[k]t$ are integrated cases of the above summands.

Hence, each summand of *n* is also a summand of $n'$. Vice versa, each summand of $n'$ is also a summand of *n*. In other words, $n=_{AC} n'$.

Finally, let the reversible process terms *s* and *t* contains +, $\cdot$, ${\text{and}}\,|$ be FR bisimilar. The TRS is terminating modulo *AC* of the +, so it reduces *s* and *t* to normal forms *n* and $n'$, respectively. Since the rewrite rules and equivalence modulo *AC* of the + can be derived from the axioms, $s=n$ and $t=n'$. Soundness of the axioms then yields $s\underline{\leftrightarrow }^{fr} n$ and $t\underline{\leftrightarrow }^{fr} n'$, so $n\underline{\leftrightarrow }^{fr} s\underline{\leftrightarrow }^{fr} t\underline{\leftrightarrow }^{fr} n'$. We showed that $n\underline{\leftrightarrow }^{fr} n'$ implies $n=_{AC}n'$. Hence, $s=n=_{AC} n'=t$.

(2) We prove the completeness of the axioms involve the parallel operator $\parallel$ and the communication merge $\between$.

The axioms RP1 and RC8-RC17 are turned into rewrite rules, by directing them from left to right.

$$\begin{aligned}&x\parallel y \rightarrow x\mid y + x\between y\\&\upsilon \between \omega \rightarrow \gamma (\upsilon ,\omega )\\&\upsilon [m]\between \omega [m] \rightarrow \gamma (\upsilon ,\omega )[m]\\&\upsilon \between (\omega \cdot y) \rightarrow \gamma (\upsilon ,\omega )\cdot y\\&\upsilon [m]\between (\omega [m]\cdot y) \rightarrow \gamma (\upsilon ,\omega )[m]\cdot y\\&(\upsilon \cdot x)\between \omega \rightarrow \gamma (\upsilon ,\omega )\cdot x\\&(\upsilon [m]\cdot x)\between \omega [m] \rightarrow \gamma (\upsilon ,\omega )[m]\cdot x\\&(\upsilon \cdot x)\between (\omega \cdot y) \rightarrow \gamma (\upsilon ,\omega )\cdot (x\parallel y)\\&(\upsilon [m]\cdot x)\between (\omega [m]\cdot y) \rightarrow \gamma (\upsilon ,\omega )[m]\cdot (x\parallel y)\\&(x + y)\between z \rightarrow x\between z + y\between z\\&x\between (y+z) \rightarrow x\between y + x\between z\\ \end{aligned}$$

Then these rewrite rules are applied to the above reversible process terms modulo *AC* of the +.

We let the weight function

$$\begin{aligned}&weight(\upsilon )\triangleq 2\\&weight(\upsilon [m])\triangleq 2\\&weight(s+t)\triangleq weight(s)+weight(t)\\&weight(s\cdot t)\triangleq weight(s)^3\cdot weight(t)^3\\&weight(s\mid t)\triangleq weight(s)^2\cdot weight(t)^2\\&weight(s\between t)\triangleq weight(s)^2\cdot weight(t)^2\\&weight(s\parallel t)\triangleq 2\cdot (weight(s)^2\cdot weight(t)^2) +1. \end{aligned}$$

We can see that the TRS is terminating modulo *AC* of the +.

We prove that normal forms n do not contain occurrences of the remaining two parallel operators $\parallel$ and $\between$. The proof is based on induction with respect to the size of the normal form *n*.

- If *n* is an atomic action, then it does not contain any parallel operators.
- Suppose $n =_{AC} s + t$ or $n =_{AC} s\cdot t$ or $n=_{AC}s\mid t$. Then by induction the normal forms *s* and *t* do not contain $\parallel$ and $\between$, so that *n* does not contain $\parallel$ and $\between$ either.
- *n* cannot be of the form $s\parallel t$, because in that case the directed version of RP1 would apply to it, contradicting the fact that n is a normal form.
- Suppose $n =_{AC} s\between t$. By induction the normal forms *s* and *t* do not contain $\parallel$ and $\between$. We can distinguish the possible forms of *s* and *t*, which all lead to the conclusion that one of the directed versions of RC8-RC17 can be applied to *n*. We conclude that *n* cannot be of the form $s\between t$.

Hence, normal forms do not contain occurrences of parallel operators $$\parallel $$ and $$\between $$. In other words, normal forms only contains $$+, \cdot $$$$\text{and }\,| $$.

Finally, let the reversible process terms *s* and *t* be FR bisimilar. The TRS is terminating modulo *AC* of the +, so it reduces *s* and *t* to normal forms *n* and $n'$, respectively. Since the rewrite rules and equivalence modulo *AC* of the + can be derived from the axioms, $s=n$ and $t=n'$. Soundness of the axioms then yields $s\underline{\leftrightarrow }^{fr} n$ and $t\underline{\leftrightarrow }^{fr} n'$, so $n\underline{\leftrightarrow }^{fr} s\underline{\leftrightarrow }^{fr} t\underline{\leftrightarrow }^{fr} n'$. We showed that $n\underline{\leftrightarrow }^{fr} n'$ implies $n=_{AC}n'$. Hence, $s=n=_{AC} n'=t$. $\square$

### Deadlock and encapsulation

A mismatch in communication of two actions $\upsilon$ and $\omega$ can cause a deadlock (nothing to do), we introduce the deadlock constant $\delta$ and extend the communication function $\gamma$ to $\gamma :C\times C\rightarrow C\cup \{\delta \}$. So, the introduction about communication merge $\between$ in the above section should be with $\gamma (\nu ,\mu )\ne \delta$. We also introduce a unary encapsulation operator $\partial _H$ for sets *H* of atomic communicating actions and their histories, which renames all actions in *H* into $\delta$. RPAP extended with deadlock constant $\delta$ and encapsulation operator $\partial _H$ is called the Algebra of Reversible Communicating Processes, which is abbreviated to ARCP.

#### Transition rules of ARCP

The encapsulation operator $\partial _H(t)$ can execute all transitions of process term *t* of which the labels are not in *H*, which is expressed by the following two forward transition rules.

$$\begin{aligned}&\frac{x\xrightarrow {\upsilon }\upsilon [m]}{\partial _H(x)\xrightarrow {\upsilon }\upsilon [m]}\quad \quad \upsilon \notin H\\&\frac{x\xrightarrow {\upsilon }x'}{\partial _H(x)\xrightarrow {\upsilon }\partial _H(x')}\quad \quad \upsilon \notin H. \end{aligned}$$

The reverse rules are as follows.

$$\begin{aligned}&\frac{x\mathop {\twoheadrightarrow }\limits ^{\upsilon [m]}\upsilon }{\partial _H(x)\mathop {\twoheadrightarrow }\limits ^{\upsilon [m]}\upsilon }\quad \quad \upsilon [m]\notin H\\&\frac{x\mathop {\twoheadrightarrow }\limits ^{\upsilon [m]}x'}{\partial _H(x)\mathop {\twoheadrightarrow }\limits ^{\upsilon [m]}\partial _H(x')}\quad \quad \upsilon [m]\notin H. \end{aligned}$$

##### **Theorem 14**

*ARCP is a conservative extension of RPAP.*

##### *Proof*

Since the TSS of RPAP is source-dependent, and the transition rules for encapsulation operator $\partial _H$ contain only a fresh operator in their source, so the TSS of ARCP is a conservative extension of that of RPAP. That means that ARCP is a conservative extension of RPAP. $\square$

##### **Theorem 15**

*FR bisimulation equivalence is a congruence with respect to ARCP.*

##### *Proof*

The TSSs for ARCP and RPAP are all in panth format, so FR bisimulation equivalence that they induce is a congruence. $\square$

#### Axiomatization for ARCP

The axioms for ARCP are shown in Table 3.

**Table 3:** Axioms for ARCP

No.	Axiom
RA6	$x+\delta = x$
RA7	$\delta \cdot x = \delta$
RA8	$x \cdot \delta = \delta$
RD1	$\upsilon \notin H\quad \partial _H(\upsilon ) = \upsilon$
RD2	$\upsilon [m]\notin H\quad \partial _H(\upsilon [m]) = \upsilon [m]$
RD3	$\upsilon \in H\quad \partial _H(\upsilon ) = \delta$
RD4	$\upsilon [m]\in H\quad \partial _H(\upsilon [m]) = \delta$
RD5	$\partial _H(\delta ) = \delta$
RD6	$\partial _H(x+y)=\partial _H(x)+\partial _H(y)$
RD7	$\partial _H(x\cdot y)=\partial _H(x)\cdot \partial _H(y)$
RD8	$\partial _H(x\mid y)=\partial _H(x)\mid \partial _H(y)$
RP8	$\delta \mid x=\delta$
RP9	$x\mid \delta =\delta$
RC18	$\delta \between x = \delta$
RC19	$x\between \delta = \delta$

The soundness and completeness theorems are following.

##### **Theorem 16**

${\mathcal {E}}_{\text {ARCP}}$*is sound for ARCP modulo FR bisimulation equivalence.*

##### *Proof*

Since FR bisimulation is both an equivalence and a congruence for ARCP, only the soundness of the first clause in the definition of the relation = is needed to be checked. That is, if $s=t$ is an axiom in ${\mathcal {E}}_{\text {ARCP}}$ and $\sigma$ a closed substitution that maps the variable in *s* and *t* to reversible process terms, then we need to check that $\sigma (s)\underline{\leftrightarrow }^{fr}\sigma (t)$.

We only provide some intuition for the soundness of the axioms in Table 3.

- RA6 says that the deadlock $\delta$ displays no behaviour, so that in a process term $s + \delta$ the summand $\delta$ is redundant.
- RA7-RA8, RP8-RP9, RC18-RC19 say that the deadlock $\delta$ blocks all behaviour.
- RD1-RD5 are the defining axioms for the encapsulation operator $\partial _H$.
- RD6-RD8 say that in $\partial _H(t)$, all transitions of *t* labelled with atomic actions from H are blocked.

These intuitions can be made rigorous by means of explicit FR bisimulation relations between the left- and right-hand sides of closed instantiations of the axioms in Table 3. Hence, all such instantiations are sound modulo FR bisimulation equivalence. $\square$

##### **Theorem 17**

${\mathcal {E}}_{\text {ARCP}}$*is complete for ARCP modulo FR bisimulation equivalence.*

##### *Proof*

To prove that ${\mathcal {E}}_{\text {ARCP}}$ is complete for ARCP modulo FR bisilumation equivalence, it means that $s\underline{\leftrightarrow }^{fr} t$ implies $s=t$.

The axioms RA6-RA8, RD1-RD8, RP8-RP9, RC18-RC19 are turned into rewrite rules, by directing them from left to right. The resulting TRS is applied to process terms in RPAP modulo AC of the +.

Then these rewrite rules are applied to the above reversible process terms modulo *AC* of the +.

We let the weight function

$$\begin{aligned}&weight(\delta )\triangleq 2\\&weight(\partial _H(s))\triangleq 2^{weight(s)}. \end{aligned}$$

We can see that the TRS is terminating modulo *AC* of the +.

We prove that normal forms n do not contain occurrences of $\partial _H$. The proof is based on induction with respect to the size of the normal form *n*.

- If $s\equiv a$, then the directed version of RA6-RA8 applies to $\partial _H(s)$.
- If $s\equiv \delta$, then the directed version of RD5 applies to $\partial _H(s)$.
- If $s=_{AC} t + t'$, then the directed version of RD6 applies to $\partial _H(s)$.
- If $s=_{AC} t \cdot t'$, then the directed version of RD7 applies to $\partial _H(s)$.
- If $s=_{AC} t \mid t'$, then the directed version of RD8 applies to $\partial _H(s)$.

Hence, normal forms do not contain occurrences of $$\partial _H$$. In other words, normal forms only contains $$+, \cdot $$$$\text{and }\,|$$.

Finally, let the reversible process terms *s* and *t* be FR bisimilar. The TRS is terminating modulo *AC* of the +, so it reduces *s* and *t* to normal forms *n* and $n'$, respectively. Since the rewrite rules and equivalence modulo *AC* of the + can be derived from the axioms, $s=n$ and $t=n'$. Soundness of the axioms then yields $s\underline{\leftrightarrow }^{fr} n$ and $t\underline{\leftrightarrow }^{fr} n'$, so $n\underline{\leftrightarrow }^{fr} s\underline{\leftrightarrow }^{fr} t\underline{\leftrightarrow }^{fr} n'$. We showed that $n\underline{\leftrightarrow }^{fr} n'$ implies $n=_{AC}n'$. Hence, $s=n=_{AC} n'=t$. $\square$

## Recursion

To capture infinite computing, recursion is introduced in this section. In ARCP, because parallel branches cannot be merged, the static parallel $\text{operator }\,|$ is a fundamental operator like + and $\cdot$ and cannot be replaced by + and $\cdot$. To what extent the existence $\text{of} \,|$ will influence the recursion theory, is a topic for our future research. In this section, we discuss recursion in reversible computation based on ARCP without the static parallel ${\text{operator}}\,|$ denoted as ARCP-RP, the corresponding axiomatization is denoted as ${\mathcal {E}}_{\text {ARCP}}-$RP2–RP9. For recursion and abstraction, it is reasonable to do extensions based on ARCP-RP (ARCP without static parallel ${\text{operator}}\,|$). Because in reversible computation, all choice branches are retained and can execute simultaneously. The choice operator + and the static parallel ${\text{operator}}\,|$ have the similar behaviors, so the static parallel operator can be naturally removed from ARCP.

In the following, *E*, *F*, *G* are guarded linear recursion specifications, *X*, *Y*, *Z* are recursive variables. We first introduce several important concepts, which come from Fokkink (2007).

### **Definition 39**

(*Recursive specification*) A recursive specification is a finite set of recursive equations

$$\begin{aligned} X_1&=  {} t_1(X_1,\ldots ,X_n)\\&\ldots \\ X_n& = {} t_n(X_1,\ldots ,X_n) \end{aligned}$$

where the left-hand sides of $X_i$ are called recursion variables, and the right-hand sides $t_i(X_1,\ldots ,X_n)$ are reversible process terms in ARCP with possible occurrences of the recursion variables $X_1,\ldots ,X_n$.

### **Definition 40**

(*Solution*) Processes $p_1,\ldots ,p_n$ are a solution for a recursive specification $\{X_i=t_i(X_1,\ldots ,X_n)|i\in \{1,\ldots ,n\}\}$ (with respect to FR bisimulation equivalence) if $p_i\underline{\leftrightarrow }^{fr}t_i(p_1,\ldots ,p_n)$ for $i\in \{1,\ldots ,n\}$.

### **Definition 41**

(*Guarded recursive specification*) A recursive specification

$$\begin{aligned} X_1& = {} t_1(X_1,\ldots ,X_n)\\&\ldots \\ X_n& = {} t_n(X_1,\ldots ,X_n) \end{aligned}$$

is guarded if the right-hand sides of its recursive equations can be adapted to the form by applications of the axioms in ${\mathcal {E}}_{\text {ARCP}}-$RP2–RP9 and replacing recursion variables by the right-hand sides of their recursive equations,

$$ a_1\cdot s_1(X_1,\ldots ,X_n)+\cdots +a_k\cdot s_k(X_1,\ldots ,X_n)+b_1+\cdots +b_l, $$

where $a_1,\ldots ,a_k,b_1,\ldots ,b_l\in A$, and the sum above is allowed to be empty, in which case it represents the deadlock $\delta$.

### **Definition 42**

(*Linear recursive specification*) A recursive specification is linear if its recursive equations are of the form

$$\begin{aligned} a_1X_1+\cdots +a_kX_k+b_1+\cdots +b_l \end{aligned}$$

where $a_1,\ldots ,a_k,b_1,\ldots ,b_l\in A$, and the sum above is allowed to be empty, in which case it represents the deadlock $\delta$.

### Transition rules of guarded recursion

For a guarded recursive specifications *E* with the form

$$\begin{aligned} X_1& = {} t_1(X_1,\ldots ,X_n)\\&\ldots \\ X_n& = {} t_n(X_1,\ldots ,X_n) \end{aligned}$$

the behavior of the solution $\langle X_i|E\rangle$ for the recursion variable $X_i$ in *E*, where $i\in \{1,\ldots ,n\}$, is exactly the behavior of their right-hand sides $t_i(X_1,\ldots ,X_n)$, which is captured by the following two forward transition rules.

$$\begin{aligned}&\frac{t_i(\langle X_1|E\rangle ,\ldots ,\langle X_n|E\rangle )\xrightarrow {\upsilon }\upsilon [m]}{\langle X_i|E\rangle \xrightarrow {\upsilon }\upsilon [m]}\\&\frac{t_i(\langle X_1|E\rangle ,\ldots ,\langle X_n|E\rangle )\xrightarrow {\upsilon } y}{\langle X_i|E\rangle \xrightarrow {\upsilon } y}. \end{aligned}$$

And the corresponding reverse transition rules follow.

$$\begin{aligned} \frac{t_i(\langle X_1|E\rangle ,\ldots ,\langle X_n|E\rangle )\mathop {\twoheadrightarrow }\limits ^{\upsilon [m]}\upsilon }{\langle X_i|E\rangle \mathop {\twoheadrightarrow }\limits ^{\upsilon [m]}\upsilon }\\ \frac{t_i(\langle X_1|E\rangle ,\ldots ,\langle X_n|E\rangle )\mathop {\twoheadrightarrow }\limits ^{\upsilon [m]} y}{\langle X_i|E\rangle \mathop {\twoheadrightarrow }\limits ^{\upsilon [m]} y}. \end{aligned}$$

#### **Theorem 18**

*ARCP-RP with guarded recursion is a conservative extension of ARCP-RP.*

#### *Proof*

Since the TSS of ARCP-RP is source-dependent, and the transition rules for guarded recursion contain only a fresh constant in their source, so the TSS of ARCP-RP with guarded recursion is a conservative extension of that of ARCP-RP. $\square$

#### **Theorem 19**

*FR bisimulation equivalence is a congruence with respect to ARCP-RP with guarded recursion.*

#### *Proof*

The TSSs for guarded recursion and ARCP-RP are all in panth format, so FR bisimulation equivalence that they induce is a congruence. $\square$

### Axiomatization for guarded recursion

The recursive definition principle (RDP) and the RSP (Recursive Specification Principle) are shown in Table 4.

**Table 4:** Recursive definition principle and recursive specification principle

No.	Axiom
RDP	$\langle X_i|E\rangle = t_i(\langle X_1|E,\ldots ,X_n|E\rangle )\quad \quad (i\in \{1,\ldots ,n\})$
RSP	if $y_i=t_i(y_1,\ldots ,y_n)$ for $i\in \{1,\ldots ,n\}$, then $y_i=\langle X_i|E\rangle \quad \quad (i\in \{1,\ldots ,n\})$

#### **Theorem 20**

${\mathcal {E}}_{\text {ARCP}}-$*RP2–RP9 + RDP + RSP is sound for ARCP-RP with guarded recursion modulo FR bisimulation equivalence*.

#### *Proof*

Since FR bisimulation is both an equivalence and a congruence for ARCP-RP with guarded recursion, only the soundness of the first clause in the definition of the relation = is needed to be checked. That is, if $s=t$ is an axiom in ${\mathcal {E}}_{\text {ARCP}}-$RP2–RP9 + RDP + RSP and $\sigma$ a closed substitution that maps the variable in *s* and *t* to reversible process terms, then we need to check that $\sigma (s)\underline{\leftrightarrow }^{fr}\sigma (t)$.

We only provide some intuition for the soundness of RDP and RSP in Table 4.

- Soundness of RDP follows immediately from the two transition rules for guarded recursion, which express that $\langle X_i|E\rangle$ and $t_i(\langle X_1|E\rangle ,\ldots ,\langle X_n|E\rangle )$ have the same initial transitions for $i\in \{1,\ldots ,n\}$.
- Soundness of RSP follows from the fact that guarded recursive specifications have only one solution modulo FR bisimulation equivalence.

These intuitions can be made rigorous by means of explicit FR bisimulation relations between the left- and right-hand sides of RDP and closed instantiations of RSP in Table 4. $\square$

#### **Theorem 21**

${\mathcal {E}}_{\text {ARCP}}-$*RP2–RP9 + RDP + RSP is complete for ARCP-RP with linear recursion modulo FR bisimulation equivalence*.

#### *Proof*

The proof is similar to the proof of “${\mathcal {E}}_{\text {ACP}}$ + RDP + RSP is complete for ACP with linear recursion modulo bisimulation equivalence”, see reference Fokkink (2007). $\square$

Firstly, each process term $t_1$ in ARCP-RP with linear recursion is provably equal to a process term $\langle X_1|E\rangle$ with *E* a linear recursive specification:

$$\begin{aligned} t_i=a_{i1}t_{i1}+\cdots +a_{ik_i}t_{ik_i}+b_{i1}+\cdots +b_{il_i} \end{aligned}$$

for $i\in \{1,\ldots ,n\}$. Let the linear recursive specification *E* consist of the recursive equations

$$\begin{aligned} X_i=a_{i1}X_{i1}+\cdots +a_{ik_i}X_{ik_i}+b_{i1}+\cdots +b_{il_i} \end{aligned}$$

for $i\in \{1,\ldots ,n\}$. Replacing $X_i$ by $t_i$ for $i\in \{1,\ldots ,n\}$ is a solution for *E*, RSP yields $t_1=\langle X_1|E\rangle$.

Then, if $\langle X_1|E_1\rangle \underline{\leftrightarrow }^{fr}\langle Y_1|E_2\rangle$ for linear recursive specifications $E_1$ and $E_2$, then $\langle X_1|E_1\rangle =\langle Y_1|E_2\rangle$ can be proved similarly.

## Abstraction

A program has internal implementations and external behaviors. Abstraction technology abstracts away from the internal steps to check if the internal implementations really display the desired external behaviors. This makes the introduction of special silent step constant $\tau$ and the abstraction operator $\tau _I$.

Firstly, we introduce the concept of guarded linear recursive specification, which comes from Fokkink (2007).

### **Definition 43**

(*Guarded linear recursive specification*) A recursive specification is linear if its recursive equations are of the form

$$\begin{aligned} a_1X_1+\cdots +a_kX_k+b_1+\cdots +b_l \end{aligned}$$

where $$a_1,\ldots ,a_k,b_1,\ldots ,b_l\in A\cup \{\tau \}.$$

A linear recursive specification *E* is guarded if there does not exist an infinite sequence of $\tau$-transitions $\langle X|E\rangle \xrightarrow {\tau }\langle X'|E\rangle \xrightarrow {\tau }\langle X''|E\rangle \xrightarrow {\tau }\cdots$.

### Silent step

A $\tau$-transition is silent, which means that it can be eliminated from a process graph. $\tau$ is an internal step and kept silent from an external observer.

Now, the set *A* is extended to $A\cup \{\tau \}$, and $\gamma$ to $\gamma :A\cup \{\tau \}\times A\cup \{\tau \}\rightarrow A\cup \{\delta \}$, the predicate $\xrightarrow {\tau }\surd$ means a successful termination after execution of $\tau$.

#### Transition rules of silent step

$\tau$ keeps silent from an external observer, which is expressed by the following transition rules.

$$\begin{aligned} \frac{}{\tau \xrightarrow {\tau }\surd } \end{aligned}$$

Transition rules for choice composition, sequential composition and guarded linear recursion that involves $\tau$-transitions are omitted.

##### **Theorem 22**

*ARCP-RP with silent step and guarded linear recursion is a conservative extension of ARCP-RP with guarded linear recursion.*

##### *Proof*

Since (1) the TSS of ARCP-RP with guarded linear recursion is source-dependent; (2) and the transition rules for the silent step $\tau$ contain only a fresh constant in their source, (3) each transition rule for choice composition, sequential composition, or guarded linear recursion that involves $\tau$-transitions, includes a premise containing the fresh relation symbol $\xrightarrow {\tau }$ or predicate $\xrightarrow {\tau }\surd$, and a left-hand side of which all variables occur in the source of the transition rule, the TSS of ARCP-RP with silent step and guarded recursion is a conservative extension of that of ARCP-RP with guarded linear recursion. $\square$

##### **Theorem 23**

*Rooted branching FR bisimulation equivalence is a congruence with respect to ARCP-RP with silent step and guarded linear recursion.*

##### *Proof*

The TSSs for ARCP-RP with silent step and guarded linear recursion are all in RBB cool format, by incorporating the successful termination predicate $\downarrow$ in the transition rules, so rooted branching FR bisimulation equivalence that they induce is a congruence. $\square$

#### Axioms for silent step

The axioms for silent step are shown in Table 5.

**Table 5:** Axioms for silent step

No.	Axiom
RB1	$x + \tau = x$
RB2	$\tau + x = x$
RB3	$\tau \cdot x = x$
RB4	$x \cdot \tau = x$

##### **Theorem 24**

${\mathcal {E}}_{\text {ARCP}}-$*RP2–RP9 + RB1–RB4 + RDP + RSP is sound for ARCP-RP with silent step and guarded linear recursion, modulo rooted branching FR bisimulation equivalence*.

##### *Proof*

Since rooted branching FR bisimulation is both an equivalence and a congruence for ARCP-RP with silent step and guarded recursion, only the soundness of the first clause in the definition of the relation $=$ is needed to be checked. That is, if $s=t$ is an axiom in ${\mathcal {E}}_{\text {ARCP}}-$RP2–RP9 + RB1–RB4 + RDP + RSP and $\sigma$ a closed substitution that maps the variable in *s* and *t* to reversible process terms, then we need to check that $\sigma (s)\underline{\leftrightarrow }^{fr}_{rb}\sigma (t)$.

We only provide some intuition for the soundness of axioms in Table 5.

The axioms in Table 5 says that the silent step $\tau$ keep real silent in reversible processes, since all choice branches are retained in reversible computation.

This intuition can be made rigorous by means of explicit rooted branching FR bisimulation relations between the left- and right-hand sides of closed instantiations of RB1–RB4. $\square$

##### **Theorem 25**

${\mathcal {E}}_{\text {ARCP}} -$*RP2–RP9 + RB1–RB4 + RDP + RSP is complete for ARCP-RP with silent step and guarded linear recursion, modulo rooted branching FR bisimulation equivalence*.

##### *Proof*

The proof is similar to the proof of “${\mathcal {E}}_{\text {ACP}}\,+\,$B1–B2 + RDP + RSP is complete for ACP with silent step and guarded linear recursion modulo rooted branching bisimulation equivalence”, see reference Fokkink (2007).

Firstly, each process term $t_1$ in ARCP-RP with silent step and guarded linear recursion is provably equal to a process term $\langle X_1|E\rangle$ with *E* a guarded linear recursive specification:

$$\begin{aligned} t_i=a_{i1}t_{i1}+\cdots +a_{ik_i}t_{ik_i}+b_{i1}+\cdots +b_{il_i} \end{aligned}$$

for $i\in \{1,\ldots ,n\}$. Let the guarded linear recursive specification *E* consist of the recursive equations

$$\begin{aligned} X_i=a_{i1}X_{i1}+\cdots +a_{ik_i}X_{ik_i}+b_{i1}+\cdots +b_{il_i} \end{aligned}$$

for $i\in \{1,\ldots ,n\}$. Replacing $X_i$ by $t_i$ for $i\in \{1,\ldots ,n\}$ is a solution for *E*, RSP yields $t_1=\langle X_1|E\rangle$.

Then, if $\langle X_1|E_1\rangle \underline{\leftrightarrow }^{fr}_{rb}\langle Y_1|E_2\rangle$ for guarded linear recursive specifications $E_1$ and $E_2$, then $\langle X_1|E_1\rangle =\langle Y_1|E_2\rangle$ can be proved similarly. $\square$

### Abstraction

Abstraction operator $\tau _I$ is used to abstract away the internal implementations. ARCP-RP extended with silent step $\tau$ and abstraction operator $\tau _I$ is denoted by $\text {ARCP-RP}_{\tau }$.

#### Transition rules of abstraction operator

Abstraction operator $\tau _I(t)$ renames all labels of transitions of *t* that are in the set *I* into $\tau$, which is captured by the following four forward transition rules and reverse transition rules.

$$\begin{aligned}&\frac{x\xrightarrow {\upsilon }\upsilon [m]}{\tau _I(x)\xrightarrow {\upsilon }\upsilon [m]}\quad \upsilon \notin I\quad \quad \frac{x\xrightarrow {\upsilon }x'}{\tau _I(x)\xrightarrow {\upsilon }\tau _I(x')}\quad \upsilon \notin I\\&\frac{x\xrightarrow {\upsilon }\upsilon [m]}{\tau _I(x)\xrightarrow {\tau }\surd }\quad \upsilon \in I\quad \quad \frac{x\xrightarrow {\upsilon }x'}{\tau _I(x)\xrightarrow {\tau }\tau _I(x')}\quad \upsilon \in I.\\&\frac{x\mathop {\twoheadrightarrow }\limits ^{\upsilon [m]}\upsilon }{\tau _I(x)\mathop {\twoheadrightarrow }\limits ^{\upsilon [m]}\upsilon }\quad \upsilon [m]\notin I\quad \quad \frac{x\mathop {\twoheadrightarrow }\limits ^{\upsilon [m]}x'}{\tau _I(x)\mathop {\twoheadrightarrow }\limits ^{\upsilon [m]}\tau _I(x')}\quad \upsilon [m]\notin I\\&\frac{x\mathop {\twoheadrightarrow }\limits ^{\upsilon [m]}\upsilon }{\tau _I(x)\mathop {\twoheadrightarrow }\limits ^{\tau }\surd }\quad \upsilon [m]\in I\quad \quad \frac{x\mathop {\twoheadrightarrow }\limits ^{\upsilon [m]}x'}{\tau _I(x)\mathop {\twoheadrightarrow }\limits ^{\tau }\tau _I(x')}\quad \upsilon [m]\in I. \end{aligned}$$

##### **Theorem 26**

$\text {ARCP-RP}_{\tau }$*with guarded linear recursion is a conservative extension of ARCP-RP with silent step and guarded linear recursion.*

##### *Proof*

Since (1) the TSS of ARCP-RP with silent step and guarded linear recursion is source-dependent; (2) and the transition rules for the abstraction operator contain only a fresh $\tau _I$ in their source, the TSS of $\text {ARCP-RP}_{\tau }$ with guarded linear recursion is a conservative extension of that of ARCP-RP with silent step and guarded linear recursion. $\square$

##### **Theorem 27**

*Rooted branching FR bisimulation equivalence is a congruence with respect to*$\text {ARCP-RP}_{\tau }$*with guarded linear recursion*.

##### *Proof*

The TSSs for $\text {ARCP-RP}_{\tau }$ with guarded linear recursion are all in RBB cool format, by incorporating the successful termination predicate $\downarrow$ in the transition rules, so rooted branching FR bisimulation equivalence that they induce is a congruence. $\square$

#### Axiomatization for abstraction operator

The axioms for abstraction operator are shown in Table 6.

**Table 6:** Axioms for abstraction operator

No.	Axiom
RTI1	$\upsilon \notin I \quad \tau _I(\upsilon )=\upsilon$
RTI2	$\upsilon \in I \quad \tau _I(\upsilon )=\tau$
RTI3	$\upsilon [m]\notin I \quad \tau _I(\upsilon [m])=\upsilon [m]$
RTI4	$\upsilon [m]\in I \quad \tau _I(\upsilon [m])=\tau$
RTI5	$\tau _I(\delta )=\delta$
RTI6	$\tau _I(x+y)=\tau _I(x)+\tau _I(y)$
RTI7	$\tau _I(x\cdot y)=\tau _I(x)\cdot \tau _I(y)$

Before we introduce the cluster fair abstraction rule, the concept of cluster is recaptured from Fokkink (2007).

##### **Definition 44**

(*Cluster*) Let *E* be a guarded linear recursive specification, and $I\subseteq A$. Two recursion variable *X* and *Y* in *E* are in the same cluster for *I* if and only if there exist sequences of transitions $\langle X|E\rangle \xrightarrow {b_1}\cdots \xrightarrow {b_m}\langle Y|E\rangle$ and $\langle Y|E\rangle \xrightarrow {c_1}\cdots \xrightarrow {c_n}\langle X|E\rangle$, where $b_1,\ldots ,b_m,c_1,\ldots ,c_n\in I\cup \{\tau \}$.

*a* or *aX* is an exit for the cluster *C* if and only if: (1) *a* or *aX* is a summand at the right-hand side of the recursive equation for a recursion variable in *C*, and (2) in the case of *AX*, either $A\notin I\cup \{\tau \}$ or $X\notin C$ (Table 7).

**Table 7:** Cluster fair abstraction rule

No.	Axiom
CFAR	If *X* is in a cluster for *I* with exits $\{\upsilon _1Y_1,\ldots ,\upsilon _mY_m,\omega _1,\ldots ,\omega _n\}$,
	then $\tau \cdot \tau _I(\langle X|E\rangle )=\tau \cdot \tau _I(\upsilon _1\langle Y_1|E\rangle ,\ldots ,\upsilon _m\langle Y_m|E\rangle ,\omega _1,\ldots ,\omega _n)$

##### **Theorem 28**

${\mathcal {E}}_{\text {ARCP-RP}_{\tau }}$*+ RSP + RDP + CFAR is sound for*$\text {ARCP-RP}_{\tau }$*with guarded linear recursion, modulo rooted branching FR bisimulation equivalence.*

##### *Proof*

Since rooted branching FR bisimulation is both an equivalence and a congruence for $\text {ARCP-RP}_{\tau }$ with guarded linear recursion, only the soundness of the first clause in the definition of the relation = is needed to be checked. That is, if $s=t$ is an axiom in ${\mathcal {E}}_{\text {ARCP-RP}_{\tau }}$ + RSP + RDP + CFAR and $\sigma$ a closed substitution that maps the variable in *s* and *t* to reversible process terms, then we need to check that $\sigma (s)\underline{\leftrightarrow }^{fr}_{rb}\sigma (t)$.

We only provide some intuition for the soundness of axioms in Table 6.

- RTI1–RTI5 are the defining equations for the abstraction operator $\tau _I$: RTI2 and RTI4 says that it renames atomic actions from I into $\tau$, while RTI1, RTI3, RTI5 say that it leaves atomic actions outside I and the deadlock $\delta$ unchanged.
- RTI6–RTI7 say that in $\tau _I(t)$, all transitions of *t* labelled with atomic actions from *I* are renamed into $\tau$.

This intuition can be made rigorous by means of explicit rooted branching FR bisimulation relations between the left- and right-hand sides of closed instantiations of RTI1–RTI7. $\square$

##### **Theorem 29**

${\mathcal {E}}_{\text {ARCP-RP}_{\tau }}$*+ RSP + RDP + CFAR is complete for*$\text {ARCP-RP}_{\tau }$*with guarded linear recursion, modulo rooted branching FR bisimulation equivalence.*

##### *Proof*

The proof is similar to the proof of “${\mathcal {E}}_{\text {ACP}_{\tau }}$ RDP + RSP +CFAR is complete for $\text {ACP}_{\tau }$ with guarded linear recursion modulo rooted branching bisimulation equivalence”, see reference Fokkink (2007).

Firstly, each process term $t_1$ in $\text {ARCP-RP}_{\tau }$ with guarded linear recursion is provably equal to a process term $\langle X_1|E\rangle$ with *E* a guarded linear recursive specification.

Then, if $\langle X_1|E_1\rangle \underline{\leftrightarrow }^{fr}_{rb}\langle Y_1|E_2\rangle$ for guarded linear recursive specifications $E_1$ and $E_2$, then $\langle X_1|E_1\rangle =\langle Y_1|E_2\rangle$ can be proved similarly. $\square$

## Verification for business protocols with compensation support

RACP has many applications, for example, it can be used in verification for business protocols with compensation support. Since a business protocol is usually cross organizational boundaries and survives for a long period of times. The failure of a business protocol can be remedied by a series of compensation operations. A business protocol with compensation support means that each atomic operations in the business protocol is corresponding to an atomic compensation operation, and the computation logic of the business protocol can be reversed.

**Fig. 1:**
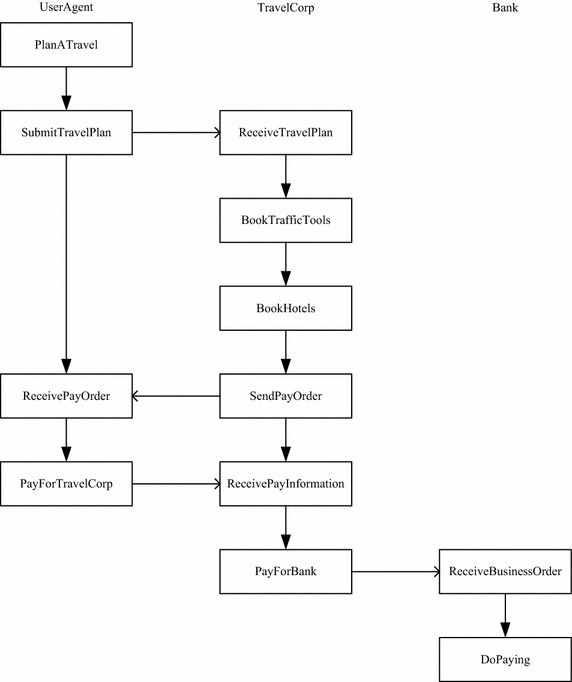
An example of business protocol

We take an example of business protocols as Fig. 1 shows. The process of the example is following, in which the user plans a travel by use of a user agent UserAgent.

1. The user plans a travel on UserAgent.
2. He/she submits the travel plan to the travel corporation TravelCorp via UserAgent.
3. TravelCorp receives the travel plan.
4. It books traffic tools and hotels according to the travel plan.
5. It sends the pay order to UserAgent.
6. UserAgent receives the pay order.
7. UserAgent sends the pay information to TravelCorp.
8. TravelAgent receives the pay information.
9. TravelAgent sends the business order to the Bank.
10. The Bank receives the business order and does paying.

### Generating the reverse (compensation) graph

The above business protocol as Fig. 1 shows can be expressed by the following reversible process term.

$$\begin{aligned} & PlanATravel \cdot SubmitTravelPlan \cdot ReceivePayOrder \cdot PayForTravelCorp \between \\ &ReceiveTravelPlan \cdot BookTrafficTools \cdot BookHotels \cdot SendPayOrder \cdot ReceivePayInformation \cdot\\ &PayForBank \between ReceiveBusinessOrder \cdot DoPaying\end{aligned}$$.

We define the following communication functions.

$$\begin{aligned}&\gamma (SubmitTravelPlan,ReceiveTravelPlan)\triangleq c_{TravelPlan}\\&\gamma (SendPayOrder,ReceivePayOder)\triangleq c_{PayOrder}\\&\gamma (PayForTravelCorp,ReceivePayInformation)\triangleq c_{PayInformation}\\&\gamma (PayForBank,ReceiveBusinessOrder)\triangleq c_{BusinessOrder} \end{aligned}$$

After the successful forward execution of the above process term, the following reversible process term can be obtained.

$$PlanATravel[m_1] \cdot c_{TravelPlan}[m_2] \cdot BookTrafficTools[m_3] \cdot BookHotels[m_4] \cdot c_{PayOrder}[m_5] \cdot c_{PayInformation}[m_6] \cdot c_{BusinessOrder}[m_7] \cdot DoPaying[m_8]$$.

After the successful reverse execution (Compensation) the above process term, the original process term can be obtained.

### Verification for business protocols with compensation support

RACP can be used in correctness verification under the framework of reversible computation for business protocols with compensation support.

**Fig. 2:**
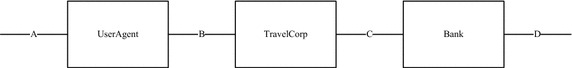
Abstractions for the example of business protocol

In Fig. 1, let UserAgent, TravelCorp and Bank be a system *UTB* and let interactions between UserAgent, TravelCorp and Bank be internal actions. *UTB* receives external input $D_i$ through channel *A* by communicating action $receive_A(D_i)$ and sends results $D_o$ through channel *D* by communicating action $send_D(D_o)$, as Fig. 2 shows.

Then the state transition of UserAgent can be described by RACP as follows.

$$\begin{aligned}&U=\sum _{D_i\in \varDelta _i}receive_A(D_i)\cdot U_1\\&U_1=PlanATravel\cdot U_2\\&U_2=SubmitTravelPlan\cdot U_3\\&U_3=ReceivePayOrder\cdot U_4\\&U_4=PayForTravelCorp\cdot U \end{aligned}$$

where $\varDelta _i$ is the collection of the input data.

The state transition of TravelAgent can be described by RACP as follows.

$$\begin{aligned} T& = {} ReceiveTravelPlan\cdot T_1\\ T_1& = {} BookTrafficTools\cdot T_2\\ T_2& = {} BookHotels\cdot T_3\\ T_3& = {} SendPayOrder\cdot T_4\\ T_4& = {} ReceivePayInformation\cdot T_5\\ T_5& = {} PayForBank\cdot T \end{aligned}$$

And the state transition of Bank can be described by RACP as follows.

$$\begin{aligned} B& = {} ReceiveBusinessOrder\cdot B_1\\ B_1& = {} DoPaying\cdot B_2\\ B_2& = {} \sum _{D_o\in \varDelta _o}send_D(D_o)\cdot B \end{aligned}$$

where $\varDelta _o$ is the collection of the output data.

We define the following communication functions.

$$\begin{aligned}&\gamma (SubmitTravelPlan,ReceiveTravelPlan)\triangleq c_{TravelPlan}\\&\gamma (SendPayOrder,ReceivePayOder)\triangleq c_{PayOrder}\\&\gamma (PayForTravelCorp,ReceivePayInformation)\triangleq c_{PayInformation}\\&\gamma (PayForBank,ReceiveBusinessOrder)\triangleq c_{BusinessOrder} \end{aligned}$$

Let *U*, *T* and *B* in parallel, then the system *UTB* can be represented by the following process term.

$$\begin{aligned} \tau _I(\partial _H(U\parallel T\parallel B)) \end{aligned}$$

where $$H=\{SubmitTravelPlan,ReceiveTravelPlan,SendPayOrder,ReceivePayOder, PayForTravelCorp,ReceivePayInformation,PayForBank,ReceiveBusinessOrder\}$$ and $$I=\{c_{TravelPlan}, c_{PayOrder}, c_{PayInformation}, c_{BusinessOrder}, BookTrafficTools, BookHotels, DoPaying\}$$.

Then we get the following conclusion.

#### **Theorem 30**

*The business protocol as Fig.* 2*shows*$\tau _I(\partial _H(U\parallel T \parallel B))$*exhibits desired external behaviors under the framework of reversible computation.*

#### *Proof*

$$\begin{aligned} \partial _H(U\parallel T \parallel B)& = {} \sum _{D_i\in \varDelta _i}receive_A(D_i)\cdot \partial _H(U_1\parallel T \parallel B)\\ \partial _H(U_1\parallel T \parallel B)& = {} PlanATravel\cdot \partial _H(U_2\parallel T \parallel B)\\ \partial _H(U_2\parallel T \parallel B)& = {} c_{TravelPlan}\cdot \partial _H(U_3\parallel T_1 \parallel B)\\ \partial _H(U_3\parallel T_1 \parallel B)& = {} BookTrafficTools\cdot \partial _H(U_3\parallel T_2 \parallel B)\\ \partial _H(U_3\parallel T_2 \parallel B)& = {} BookHotels\cdot \partial _H(U_3\parallel T_3 \parallel B)\\ \partial _H(U_3\parallel T_3 \parallel B)& = {} c_{PayOrder}\cdot \partial _H(U_4\parallel T_4 \parallel B)\\ \partial _H(U_4\parallel T_4 \parallel B)& = {} c_{PayInformation}\cdot \partial _H(U\parallel T_5 \parallel B)\\ \partial _H(U\parallel T_5 \parallel B)& = {} c_{BusinessOrder}\cdot \partial _H(U\parallel T \parallel B_1)\\ \partial _H(U\parallel T \parallel B_1)& = {} DoPaying\cdot \partial _H(U\parallel T \parallel B_2)\\ \partial _H(U\parallel T \parallel B_2)& = {} \sum _{D_o\in \varDelta _o}send_D(D_o)\cdot \partial _H(U\parallel T \parallel B) \end{aligned}$$

Let $\partial _H(U\parallel T \parallel B)=\langle X_1|E\rangle$, where E is the following guarded linear recursion specification:

$$\begin{aligned} \{X_1& = {} \sum _{D_i\in \varDelta _i}receive_A(D_i)\cdot X_2,X_2=PlanATravel\cdot X_3,X_3=c_{TravelPlan}\cdot X_4,\\ X_4& = {} BookTrafficTools\cdot X_5,X_5=BookHotels\cdot X_6,X_6=c_{PayOrder}\cdot X_7,\\ X_7& = {} c_{PayInformation}\cdot X_8,X_8=c_{BusinessOrder}\cdot X_9,X_9=DoPaying\cdot X_{10},X_{10}=\sum _{D_o\in \varDelta _o}send_B(D_o)\cdot X_1\} \end{aligned}$$

Then we apply abstraction operator $\tau _I$ into $\langle X_1|E\rangle$.

$$\begin{aligned} \tau _I(\langle X_1|E\rangle )& = {} \sum _{D_i\in \varDelta _i}receive_A(D_i)\cdot \tau _I(\langle X_2|E\rangle )\\& = {} \sum _{D_i\in \varDelta _i}receive_A(D_i)\cdot \tau _I(\langle X_3|E\rangle )\\& = {} \sum _{D_i\in \varDelta _i}receive_A(D_i)\cdot \tau _I(\langle X_4|E\rangle )\\& = {} \sum _{D_i\in \varDelta _i}receive_A(D_i)\cdot \tau _I(\langle X_5|E\rangle )\\& = {} \sum _{D_i\in \varDelta _i}receive_A(D_i)\cdot \tau _I(\langle X_6|E\rangle )\\& = {} \sum _{D_i\in \varDelta _i}receive_A(D_i)\cdot \tau _I(\langle X_7|E\rangle )\\& = {} \sum _{D_i\in \varDelta _i}receive_A(D_i)\cdot \tau _I(\langle X_8|E\rangle )\\& = {} \sum _{D_i\in \varDelta _i}receive_A(D_i)\cdot \tau _I(\langle X_9|E\rangle )\\& = {} \sum _{D_i\in \varDelta _i}receive_A(D_i)\cdot \tau _I(\langle X_{10}|E\rangle )\\& = {} \sum _{D_i\in \varDelta _i}\sum _{D_o\in \varDelta _o}receive_A(D_i)\cdot send_D(D_o)\cdot \tau _I(\langle X_1|E\rangle ) \end{aligned}$$

We get $\tau _I(\langle X_1|E\rangle )=\sum _{D_i\in \varDelta _i}\sum _{D_o\in \varDelta _o}receive_A(D_i)\cdot send_D(D_o)\cdot \tau _I(\langle X_1|E\rangle )$, that is, $\tau _I(\partial _H(U\parallel T \parallel B))=\sum _{D_i\in \varDelta _i}\sum _{D_o\in \varDelta _o}receive_A(D_i)\cdot send_D(D_o)\cdot \tau _I(\partial _H(U\parallel T \parallel B))$. So, the business protocol as Fig.2 shows $\tau _I(\partial _H(U\parallel T \parallel B))$ exhibits desired external behaviors. $\square$

## Extensions

One of the most fascinating characteristics is the modularity of RACP, that is, RACP can be extended easily. Through out this paper, we can see that RACP also inherents the modularity characteristics of ACP. By introducing new operators or new constants, RACP can have more properties. It provides RACP an elegant fashion to express a new property.

In this section, we take an example of renaming operators which are used to rename the atomic actions.

### Transition rules of renaming operators

Renaming operator $\rho _f(t)$ renames all actions in process term *t*, and assumes a renaming function $f:A\rightarrow A$, which is expressed by the following two forward transition rules and two reverse ones.

$$\begin{aligned}&\frac{x\xrightarrow {\upsilon }\upsilon [m]}{\rho _f(x)\xrightarrow {f(\upsilon )}f(\upsilon )[m]}\\&\frac{x\xrightarrow {\upsilon }x'}{\rho _f(x)\xrightarrow {f(\upsilon )}\rho _f(x')}\\&\frac{x\mathop {\twoheadrightarrow }\limits ^{\upsilon [m]}\upsilon }{\rho _f(x)\mathop {\twoheadrightarrow }\limits ^{f(\upsilon )[m]}f(\upsilon )}\\&\frac{x\mathop {\twoheadrightarrow }\limits ^{\upsilon [m]}x'}{\rho _f(x)\mathop {\twoheadrightarrow }\limits ^{f(\upsilon )[m]}\rho _f(x')} \end{aligned}$$

#### **Theorem 31**

$\text {ARCP-RP}_{\tau }$*with guarded linear recursion and renaming operators is a conservative extension of*$\text {ARCP-RP}_{\tau }$*with guarded linear recursion.*

#### *Proof*

Since (1) the TSS of $\text {ARCP-RP}_{\tau }$ with guarded linear recursion is source-dependent; (2) and the transition rules for the renaming operators contain only a fresh $\rho _f$ in their source, the TSS of $\text {ARCP-RP}_{\tau }$ with guarded linear recursion and renaming operators is a conservative extension of that of $\text {ARCP-RP}_{\tau }$ with guarded linear recursion. $\square$

#### **Theorem 32**

*Rooted branching FR bisimulation equivalence is a congruence with respect to*$\text {ARCP-RP}_{\tau }$*with guarded linear recursion and renaming operators.*

#### *Proof*

The TSSs for $\text {ARCP-RP}_{\tau }$ with guarded linear recursion and renaming operators are all in RBB cool format, by incorporating the successful termination predicate $\downarrow$ in the transition rules, so rooted branching FR bisimulation equivalence that they induce is a congruence. $\square$

### Axioms for renaming operators

The axioms for renaming operator is shown in Table 8.

**Table 8:** Axioms for renaming

No.	Axiom
RRN1	$\rho _f(\upsilon )=f(\upsilon )$
RRN2	$\rho _f(\upsilon [m])=f(\upsilon )[m]$
RRN3	$\rho _f(\delta )=\delta$
RRN4	$\rho _f(x+y)=\rho _f(x)+\rho _f(y)$
RRN5	$\rho _f(x\cdot y)=\rho _f(x)\cdot \rho _f(y)$

#### **Theorem 33**

${\mathcal {E}}_{\text {ARCP-RP}_{\tau }}$*+ RSP + RDP + CFAR + RRN1–RRN5 is sound for*$\text {ARCP-RP}_{\tau }$*with guarded linear recursion and renaming operators, modulo rooted branching FR bisimulation equivalence.*

#### *Proof*

Since rooted branching FR bisimulation is both an equivalence and a congruence for $\text {ARCP-RP}_{\tau }$ with guarded linear recursion and renaming operators, only the soundness of the first clause in the definition of the relation = is needed to be checked. That is, if $s=t$ is an axiom in ${\mathcal {E}}_{\text {ARCP-RP}_{\tau }}$ + RSP + RDP + CFAR + RRN1-RRN5 and $\sigma$ a closed substitution that maps the variable in *s* and *t* to reversible process terms, then we need to check that $\sigma (s)\underline{\leftrightarrow }^{fr}_{rb}\sigma (t)$.

We only provide some intuition for the soundness of axioms in Table 8.

- RRN1–RRN3 are the defining equations for the renaming operator $\rho _f$.
- RRN4–RRN5 say that in $\rho _f(t)$, the labels of all transitions of *t* are renamed by means of the mapping *f*.

This intuition can be made rigorous by means of explicit rooted branching FR bisimulation relations between the left- and right-hand sides of closed instantiations of RRN1-RRN5. $\square$

#### **Theorem 34**

${\mathcal {E}}_{\text {ARCP-RP}_{\tau }}$*+ RSP + RDP + CFAR + RRN1-RRN5 is complete for*$\text {ARCP-RP}_{\tau }$*with guarded linear recursion and renaming operators, modulo rooted branching FR bisimulation equivalence.*

#### *Proof*

It suffices to prove that each process term *t* in $\text {ARCP-RP}_{\tau }$ with guarded linear recursion and renaming operators is provably equal to a process term $\langle X|E\rangle$ with *E* a guarded linear recursive specification. Namely, then the desired completeness result follows from the fact that if $\langle X_1|E_1\rangle \underline{\leftrightarrow }^{fr}_{rb}\langle Y_1|E_2\rangle$ for guarded linear recursive specifications $E_1$ and $E_2$, then $\langle X_1|E_1\rangle =\langle Y_1|E_2\rangle$ can be derived from ${\mathcal {E}}_{\text {ARCP-RP}_{\tau }}$ + RSP + RDP + CFAR.

Structural induction with respect to process term *t* can be applied. The only new case (where RRN1-RRN5 are needed) is $t \equiv \rho _f(s)$. First assuming $s=\langle X_1|E\rangle$ with a guarded linear recursive specification *E*, we prove the case of $t=\rho _f(\langle X_1|E\rangle )$. Let *E* consists of guarded linear recursive equations

$$\begin{aligned} X_i=a_{i1}X_{i1}+\cdots +a_{ik_i}X_{ik_i}+b_{i1}+\cdots +b_{il_i} \end{aligned}$$

for $i\in {1,\ldots ,n}$. Let *F* consists of guarded linear recursive equations

$$\begin{aligned} Y_j=f(a_{i1})Y_{i1}+\cdots +f(a_{ik_i})Y_{ik_i}+f(b_{i1})+\cdots +f(b_{il_i}) \end{aligned}$$

for $$j\in {1,\ldots ,n}$$.

$$\begin{aligned}&\rho _f(\langle X_i|E\rangle ) \\ &\overset{\text {RDP}}{=} \rho _f(a_{i1}X_{i1}+\cdots +a_{ik_i}X_{ik_i}+b_{i1}+\cdots +b_{il_i})\\&\overset{\text {RRN1-RRN5}}{=} \rho _f(a_{i1})\cdot \rho _f(X_{i1})+\cdots +\rho _f(a_{ik_i})\cdot \rho _f(X_{ik_i})+\rho _f(b_{i1})+\cdots +\rho _f(b_{il_i}) \end{aligned}$$

Replacing $Y_i$ by $\rho _f(\langle X_i|E\rangle )$ for $i\in \{1,\ldots ,n\}$ is a solution for *F*. So by RSP, $\rho _f(\langle X_1|E\rangle )=\langle Y_1|F\rangle$. $\square$

## Conclusions

In this paper, we give reversible computation an axiomatic foundation called RACP. RACP can be widely used in verification of applications in reversible computation.

For recursion and abstraction, it is reasonable to do extensions based on ARCP-RP (ARCP without static parallel ${\text{operator}}\,|$). Because in reversible computation, all choice branches are retained and can execute simultaneously. The choice operator + and the static parallel ${\text{operator}}\,|$ have the similar behaviors, so the static parallel operator can be naturally removed from ARCP.

Any computable process can be represented by a process term in ACP (exactly $\text {ACP}_\tau$ with guarded linear recursion) Baeten et al. (1987). That is, ACP may have the same expressive power as Turing machine. And RACP may have the same expressive power as ACP.

Same as ACP, RACP has good modularity and can be extended easily. Although the extensions can not improve the expressive power of RACP, it still provides an elegant and convenient way to model other properties in reversible computation.
